# Lipidome Atlas of the Developing Heart Uncovers Dynamic Membrane Lipid Attributes Underlying Cardiac Structural and Metabolic Maturation

**DOI:** 10.34133/research.0006

**Published:** 2022-12-19

**Authors:** Huan Miao, Bowen Li, Zehua Wang, Jinming Mu, Yanlin Tian, Binhua Jiang, Shaohua Zhang, Xia Gong, Guanghou Shui, Sin Man Lam

**Affiliations:** ^1^ State Key Laboratory of Molecular Developmental Biology, Institute of Genetics and Developmental Biology, Chinese Academy of Sciences, Beijing 100101, China.; ^2^ University of Chinese Academy of Sciences, Beijing 100049, China.; ^3^ LipidALL Technologies Company Limited, Changzhou 213022, Jiangsu Province, China.

## Abstract

Precise metabolic rewiring during heart organogenesis underlies normal cardiac development. Herein, we utilized high-coverage, quantitative lipidomic approaches to construct lipidomic atlases of whole hearts (861 lipids; 31 classes) and mitochondria (587 lipids; 27 classes) across prenatal and postnatal developmental stages in mice. We uncovered the progressive formation of docosahexaenoyl-phospholipids and enhanced remodeling of C18:2, C20:3, and C20:4 fatty acyl moieties into cardiolipins as cardiac development progresses. A preferential flow of ceramides toward sphingomyelin biosynthesis over complex glycosphingolipid formation was also noted. Using maSigPro and GPclust algorithms, we identified a repertoire of 448 developmentally dynamic lipids and mapped their expression patterns to a library of 550 biologically relevant developmentally dynamic genes. Our combinatorial transcriptomics and lipidomics approaches identified *Hadha, Lclat1*, and *Lpcat3* as candidate molecular drivers governing the dynamic remodeling of cardiolipins and phospholipids, respectively, in heart development. Our analyses revealed that postnatal cardiolipin remodeling in the heart constitutes a biphasic process, which first accumulates polyunsaturated C78-cardiolipins prior to tetralinoleoyl cardiolipin forming the predominant species. Multiomics analyses supplemented with transmission electron microscopy imaging uncovered enhanced mitochondria–lipid droplet contacts mediated by perilipin-5. Our combinatorial analyses of multiomics data uncovered an association between mitochondrial-resident, docosahexaenoic acid-phospholipids and messenger RNA levels of proton-transporting adenosine triphosphate synthases on inner mitochondrial membranes, which adds credence to the membrane pacemaker theory of metabolism. The current findings offer lipid-centric biological insights potentially important to understanding the molecular basis of cardiac metabolic flexibility and disease pathology.

## Introduction

The heart, an indispensable organ with fascinating developmental biology, denotes the first organ to become functional in the developing mammalian embryo [1]. Improper development of the heart can result in congenital heart defects, which represent some of the most common and deadliest birth defects [2]. As the organ with the highest caloric requirement exhibiting the most robust oxidation of fatty acids [3], the adult heart retains flexibility in its choice of metabolic fuel, with approximately 60% to 80% of its energy demand being fulfilled by fatty acid oxidation, and glucose, lactate, and ketone bodies meeting the remainder [3]. Disproportionate utilization of fatty acids coupled with an excess supply in the circulating plasma can create lipid-induced cardiotoxicity. Indeed, an increasing trend toward sedentary lifestyles and calorie overconsumption has fueled a global obesity pandemic with its associated cardiometabolic sequelae. Overnutrition leads to the ectopic deposition of lipids in nonadipose tissues such as the heart, which can have serious implications on its normal physiology [4]. Cardiac lipid metabolism is intricately connected to heart physiology. Abnormal regulation of intracellular lipid metabolism impinges on mitochondrial oxidative phosphorylation, which can detrimentally afflict cardiac function [5,6].

Birth signifies a critical transition in cardiac physiology and energy metabolism. The fetal cardiac milieu is marked by low oxygen and substrate respiratory coefficient, with myocardial energy principally derived from glycolysis and lactate oxidation. Following birth, the newborn heart rapidly increases mitochondrial oxidative phosphorylation and skews energy substrate preference towards fatty acyls, increasing cardiac output in order to fulfill enhanced metabolic demands and operate against an elevated vascular resistance [7]. The metabolic transition takes approximately 7 days, which indicates that subcellular (structural) maturational events govern the transition, given that the newborn heart remains unable to substantially oxidize fatty acids despite a sharp increase in plasma fatty acid supply within hours of birth [7,8].

Cardiomyocytes, the working units of the heart, contain an abundance of mitochondria to produce energy that sustains the pumping of the heart. Cardiolipin (CL), localized at the inner mitochondrial membrane (IMM), denotes the signature phospholipid class of the mitochondria [9]. CL comprises 2 phosphatidylglycerol (PG) moieties esterified to a glycerol backbone, rendering its unique structural and functional role in the mitochondria [10]. CL is critical to maintaining the ultrastructure of mitochondrial cristae, which are membrane foldings of the IMM—the main site of oxidative phosphorylation where protein components of the electron transport chains are localized [11]. Mitochondria crista can undergo remodeling to fine-tune mitochondria function in response to cellular metabolic demands. Tafazzin catalyzes CL remodeling, producing “mature” CLs that contain tissue- and function-specific fatty acyl compositions usually different from nascent CLs at the end of the biosynthetic process. Tafazzin knockdown in mice results in abnormal cristae morphology and perturbed mitochondrial function [12]. Besides Tafazzin, other acyltransferases can also mediate the re-acylation of monolyso-cardiolipins in mammalian cells, namely, the acyl-coenzyme A (CoA):lysocardiolipin acyltransferase, the hydratase subunit A (Hadha)/tri-functional protein alpha [13,14], and the monolyso-cardiolipin acyltransferase [15], a splice variant of Hadha [16].

Elucidating the molecular and metabolic basis of heart organogenesis from prenatal to postnatal stages, thus, can help us understand better how the heart modulates metabolic flexibility later in life—an important aspect of normal cardiac physiology and homeostasis. Indeed, global transcriptomic atlases of the developing heart have been previously reported [17], even up to the resolution of single cell [18]. In contrast, a comprehensive lipidomic map of heart organogenesis has remained lacking. Membrane lipid composition affects cardiac function and physiology. For instance, a strong positive correlation was observed between docosahexaenoic acid (DHA) content of cardiac phospholipids and the heart rate of a diverse group of mammals ranging from mice to whales [19]. The membrane pacemaker theory of metabolism proposes DHA as an important (but not sole) contributor to the degree of lipid membrane polyunsaturation, which, in turn, fine-tunes molecular activities of transmembrane proteins, particularly sodium/potassium (Na^+^/K^+^) pumps on plasmalemma and proton pumps [H^+^ transporting adenosine triphosphate (ATP) synthase] on the IMMs, and exerts a deterministic effect on basal cellular metabolism [20]. Metabolic rewiring of cardiomyocytes is also a known hallmark of heart failure [21]. High-coverage quantitative lipidomics and subsequent systems investigation of developmentally dynamic lipids (DDLs) across heart development can unravel molecular candidates underlying cardiac metabolic transition across the prenatal and postnatal stages and offer potential insights into cardiac metabolic homeostasis and dysregulation in later life [22]. In addition, we leveraged on published temporal transcriptome data of the developing heart [17] and performed an integrated analysis of transcriptome and lipidome changes across the trajectory of heart development. Our integrated analyses revealed accumulation of DHA-phospholipids and increasing CL unsaturation as the key dynamic membrane lipid attributes driving structural and metabolic adaptations crucial to the maturing heart.

## Results

### Segregation of lipidomes based on cardiac structural and metabolic maturation

**Fig. 1.**
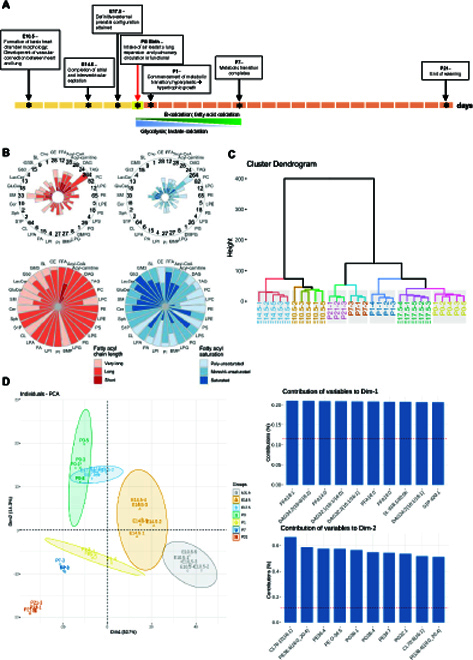
Global lipidome of the developing heart. (A) Whole hearts from 7 developmental time points along the prenatal and postnatal period of heart development were collected, which include E10.5 that marks the formation of basic heart structures (*n* = 5), E14.5 at which atrial and interventricular septation completes (*n* = 5), E17.5 for which definitive external configuration is attained (*n* = 5), P0 at birth (*n* = 4), P1 that marks the commencement of fuel substrate reliance on fatty acids over lactate (*n* = 4), P7 at which cardiac metabolic transition completes (*n* = 3), and P21 that marks the end of weaning (*n* = 3). E, embryonic; P, postnatal. (B) A total of 861 lipid species spanning 31 individual lipid classes was identified and quantitated in the heart tissue lipidome of the study. Radar diagrams illustrate the distribution of fatty acyls of different carbon atom numbers and double bond numbers in major lipid classes for P21 heart lipidome. The numbers at the circumferential boundary of the upper panel radar diagrams indicate the number of quantitated lipid species within each lipid class. Classification of carbon atom numbers: short (C < 16), long (C16 to C21), and very long (C > 22); classification of fatty acyl unsaturation (double bond number *n*): saturated (*n* = 0), mono/di-unsaturated (*n* = 1 to 2), and polyunsaturated (*n* > 3). (C) Cluster dendogram analysis of individual samples indicated high intergroup variability and low intragroup variability. (D) Principal component analysis (PCA) of individual samples with bracketed numbers indicating the percentage of total variance explained by each component. The top 10 contributing variables to each component (i.e., Dim-1 and Dim-2) were shown respectively in the bar plots on the right panel.

Seven time points (E10.5, E14.5, E17.5, P0, P1, P7, and P21) were sampled and investigated across prenatal/embryonic (E) and postnatal (P) heart development (Fig. 1A), comprising 30 animals in total. We quantitated a repertoire of 861 lipid species from 31 major lipid classes (Fig. 1B and Fig. S1). The greatest diversity in carbon atom numbers and double bond numbers of acyl chains was found in triacylglycerols (TAGs), while sphingolipid (SPL) classes including sphingomyelin (SM), ceramide (Cer), glucosylceramide (GluCer), lactosylceramide (LacCer), monosialodihexosyl gangliosides (GM3), and globotriaosylceramides (Gb3) comprise predominantly very-long-chain fatty acyls that are either saturated or monounsaturated in nature (Fig. 1B). Cluster dendogram of individual samples indicated a high degree of intragroup consistency (i.e., samples of the same developmental stage were clustered within a clade) (Fig. 1C). According to expectations, we observed that heart lipidomes at E10.5 and E14.5, which represent the periods prior to the complete formation of definitive external prenatal configuration, were more closely related to each other and distinct from the remaining stages. Stages of lipidomes from E17.5, P0, and P1, during which the overall heart configuration is attained but metabolic transition has yet to complete, were more closely related to each other but different from those of P7 and P21, which marked the completion of metabolic transition from glycolysis to enhanced reliance on mitochondrial oxidative phosphorylation of fatty acid fuel. Thus, the clustering of lipidomes essentially revealed the importance of lipids in both cardiac structural maturation and metabolic rewiring. We then deployed principal component analysis (PCA) to explore the global relationships among the samples (Fig. 1D). We found that the first dimension (Dim-1) separates the samples more based on the degree of structural development, i.e., E10.5 and E14.5 from the later stages with complete external prenatal configuration. The top 10 lipids constituting Dim-1 were predominantly saturated free fatty acids (FFAs) (FFA 14:0, FFA 16:0, and FFA 18:0) and numerous diacylglycerols (DAGs). On the other hand, the second dimension (Dim-2) separates the heart samples based on metabolic differences, i.e., E17.5 and P0 from P1, P7, and P21, since P1 marks approximately the commencement of metabolic transition following birth. Top lipids contributing to Dim-2 comprise mitochondria-resident CLs containing relatively shorter fatty acyls, CL76:12(16:1) and CL70:8(16:1), CL-biosynthetic precursors PGs, and polyunsaturated diacyl and plasmanyl phosphatidylethanolamines (PEs) comprising arachidonic fatty acyls (C20:4). These lipid changes imply that mitochondria exert important roles in the metabolic transition of the developing heart following birth and that PG and PE may participate in this process.

### Identification of DDLs

**Fig. 2.**
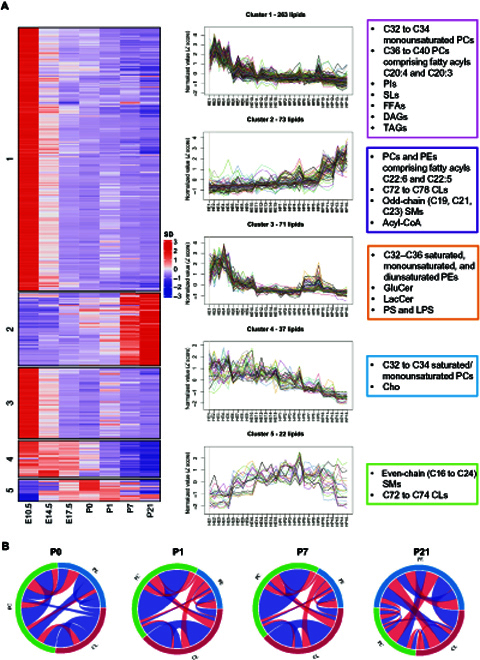
maSigPro identified 5 clusters of developmentally dynamic lipids (DDLs) in the heart. (A) Lipid species were partitioned into 5 clusters by hierarchical clustering algorithm according to their variation patterns. The left panel shows row-normalized abundance (median of replicates) of lipid species with significant temporal changes across heart development. The middle panel shows changes in individual lipid species across samples ordered by developmental time points. Five clusters of DDLs were identified across prenatal and postnatal stages of heart development. Cluster 1 (249 lipids) and cluster 3 (70 lipids) denote early DDLs (highest levels in early developmental stages), cluster 2 (71 lipids) represents late DDLs (highest levels in late developmental stages), cluster 4 (37 lipids) comprises DDLs that peaked shortly prior to birth, and cluster 5 (21 lipids) consists of DDLs that peak at birth and then decrease. Representative lipids of each cluster are boxed and illustrated on the right panel. (B) Chord diagrams illustrate changes in lipid correlations among classes of CL, PC, and PE across postnatal heart development. Correlations between lipids were calculated using Spearman’s correlation analysis in each postnatal developmental stage. Band width indicates the number of significant correlations and color indicates direction of correlation. *P* value cutoff was set at *P* < 0.05. Blue shade indicates positive correlations, while red shade indicates negative correlation between 2 connecting lipids.

We then used maSigPro to identify statistically significant differential expression profiles from the time-course lipidome data, which yielded 448 DDLs classified into 5 major clusters (Fig. 2). Clusters 1 and 3 contain “early DDLs” with peak levels at the earliest development time point (i.e., E10.5) that subsequently declined. Clusters 1 and 3 contained phosphatidylcholines (PCs) and PEs comprising relatively short (C32 to C36), monounsaturated/diunsaturated acyl chains, as well as medium-chain PCs (C36 to C40) with C20:4 and C20:3 acyl constituents. Importantly, neutral storage lipids, including TAGs, DAGs, and FFAs, as well as glycosylated SPLs like GluCer, LacCer, and sulfatides (SLs), also reside within these early DDLs clusters. Closely related to clusters 1 and 3 is cluster 4, which comprises DDLs with peak expression shortly prior to birth and then decreased subsequently. Cluster 4 contains PCs with short (C32 to C34) and saturated/monounsaturated acyl chains as well as free cholesterol (Cho). Opposed to early DDLs, cluster 2 contains “late DDLs” that gradually increased in levels across development. Interestingly, these late DDLs include PCs and PEs with docosahexaenoic (DHA) and docosapentaenoic fatty acyls, polyunsaturated C72 to C78 CLs, and odd-chain SMs. Finally, cluster 5 contains DDLs that displayed an inverted-U shape in their expression patterns, with levels peaking at birth (P0), and consists of even-chain SMs and CLs with shorter total carbon atom numbers (C72 to C74) compared to cluster 3. Changes in the patterns of DDLs, henceforth, indicate an overall massive remodeling in fatty acyl carbon number and unsaturation index towards longer, more unsaturated chains across development.

### Increased channeling of C18:2, C20:3, and C20:4 acyl substrates from PC to PE to CL

We investigated patterns of changes in fatty acyl chain lengths and double bonds across heart development using fuzzy c-means (Fig. S2). We emphasized on 5 classes of lipids that were particularly crucial to mitochondrial function, including acylcarnitines, CLs, PCs, PEs, and phosphatidylserines (PSs). As aforementioned, CLs are signature lipids of mitochondria that associate with protein components of the electron transport chain on IMM [11], while acylcarnitines are important carriers of fatty acyls into the mitochondrial space [23]. PC, PE, and PS, on the other hand, represent major phospholipid classes that supply fatty acyl substrates to CL remodeling during the structural maturation of mitochondria [24]. We observed that the levels of total acylcarnitines decreased across development, while that of total CL increased. Total PC, PE, and PS, on the other hand, did not change significantly across development (Fig. S2A). Individual lipids from each class were categorized into 4 subgroups on the right panel according to their specific patterns of changes (Fig. S2B), and it was noted that members of CL, PC, and PE displayed distinct, opposing patterns of changes across heart development. These observations indicate active remodeling of PC, PE, and CL fatty acyl constituents as heart development progresses. We then delved into the proportional changes in carbon atom numbers and double bond numbers of fatty acyl constituents under each lipid class, and noticed a general pattern of increasing carbon atom number and degree of unsaturation as heart development progresses (Fig. S2C and D). As development progresses, members of CLs, PCs, and PEs undergo increases in acyl chain length and degree of unsaturation (Fig. S2C and D) mainly attributed to increasing formation of DHA-PCs and DHA-PEs (C22:6), since species carrying C20:4 and C20:3 acyls are decreased. At the same time, acyl constituents of CLs become increasingly remodeled from C16:1 into C18:2, C20:3, and C20:4 (Fig. 2; clusters 5 and 2). As PCs and PEs denote possible sources of fatty acyl moieties for CL remodeling during heart development, we also examined changes in lipid coregulation between CL, PC, and PE based on Spearman correlations as illustrated by chord diagrams (Fig. 2B). At P0, CLs displayed little correlation with PCs and PEs. By P21, however, positive correlations (blue shade) between CLs and PEs emerged, while negative correlations (red shade) between PCs and PEs also increased (Fig. 2B). Increasingly intense correlations between long-chain C78-CLs and PEs carrying C20:3 and C20:4 acyl moieties suggest that PEs may supply these polyunsaturated acyl substrates for CL remodeling as postnatal development progresses (source data table for Fig. 2B) or that PEs and CLs may function in concert during postnatal metabolic transition. The increasingly negative correlation between PCs and PEs, on another note, indicates a possibly blunted conversion between these lipid classes in postnatal developmental stages.

### Increases in lipid membrane fluidity as cardiac development progresses

**Fig. 3.**
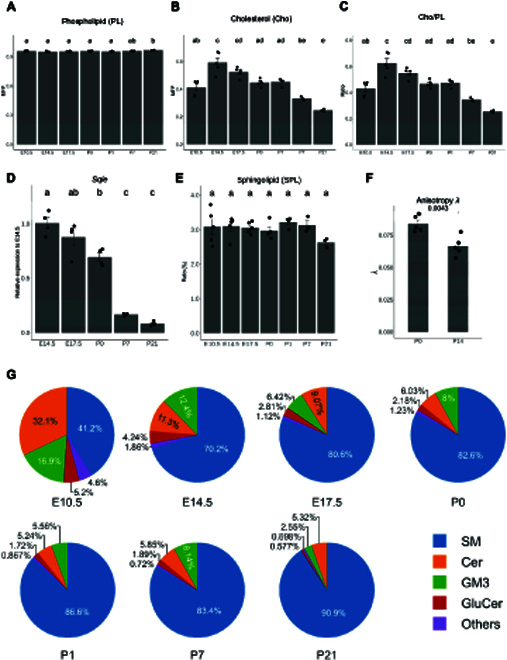
Increases in lipid membrane fluidity as cardiac development progresses. Lipid-based compositional indices indicated that lipid membrane fluidity increases during the course of heart development. Changes in lipid indices and gene expressions were compared using ANOVA with Tukey’s HSD post hoc, and statistical significance was indicated using letter-based representation of all pairwise comparisons, such that 2 groups sharing a common letter are not significantly different at *P* < 0.05. (A to C) Changes in sum of total phospholipids (PL) (A), free cholesterol (Cho) (B), and ratio of free cholesterol to total phospholipids (Cho/PL) (C) across heart development. Total PL was calculated as the sum of PC + PE + PI + PS + PA + PG + CL + LBPA + LPC + LPE + LPG + LPS + LPI + LPA. Error bars were means + standard error of the mean (SEM). (D) Changes in the relative expression of squalene epoxidase (*Sqle*) normalized to the housekeeping gene β-actin, the rate-limiting enzyme in the mevalonate pathway of Cho biosynthesis across heart development. Error bars were means + SEM. (E) Changes in the sum of total sphingolipids (SPLs) across heart development. Total SPL was calculated by the sum of SM + Cer + GluCer + GM3 + Gb3 + SL + S1P + LacCer + Sph. Error bars were means + SEM. (F) Membrane anisotropy (*λ*) of sarcolemma membranes isolated from heart tissues at P0 and P14. Error bars were means + SEM. *n* = 5 technical replicates from sarcolemma membranes pooled from 9 animals for P0 and 6 animals for P14. (G) Pie charts illustrate relative distributions of major SPL classes (by abundance), including SM, Cer, GluCer, and GM3 across heart development. SM, sphingomyelin; Cer, ceramide; GluCer, glucosylceramide; GM3, monosialo-dihexosyl ganglioside; Gb3, globotriaosylceramide; SL, sulfatide; S1P, sphingosine-1-phosphate; LacCer, lactosylceramide; Sph, sphingosine.

As compositional changes (i.e., acyl chain lengths and unsaturation index) in cardiac lipidome suggest changing lipid membrane fluidity over the course of heart development, we next surveyed several lipid indices reflective of membrane anisotropy, with particular focus on changes in the ratios of lipid raft constituents (i.e., Cho and SPLs) across development (Fig. 3). Total phospholipids (PLs) did not alter appreciably (Fig. 3A) but total Cho exhibited a constant drop from E14.5 onwards (Fig. 3B), which translates to a reducing ratio of Cho/PL (Fig. 3C) in lipid membranes as heart development progresses. The ratio of Cho/PL is a widely accepted indicator of lipid membrane fluidity [25]. In accordance with lipid profile changes, the relative expression of squalene epoxidase (*Sqle*), a rate-limiting enzyme in the mevalonate pathway of Cho biosynthesis [26], was reduced progressively from E14.5 onwards (Fig. 3D). Proportion of total SPLs, however, was maintained relatively unaltered at 3% across development, apart from a slight drop at P21 (Fig. 3E). We examined membrane anisotropy of sarcolemma membranes isolated from heart tissues at P0 and P14 using the fluorescent probe 1,6-diphenyl-1,3,4-hexatriene (DPH). Corroborating our lipidomic observations, sarcolemma membranes isolated from P14 heart tissues were more fluid (i.e., less anisotropic) than those from P0 (Fig. 3F). Despite a relatively constant level of total SPL, the proportion of major SPL classes changed appreciably across heart development, with gradual decline in GM3s accompanied by increases in SMs at P21 (Fig. 3G), suggesting an enhanced flow of Cer precursors for SM biosynthesis over complex glycosphingolipid production as cardiac development ensues.

### Enhanced mitochondria-associated neutral lipid depots during cardiac maturation

**Fig. 4.**
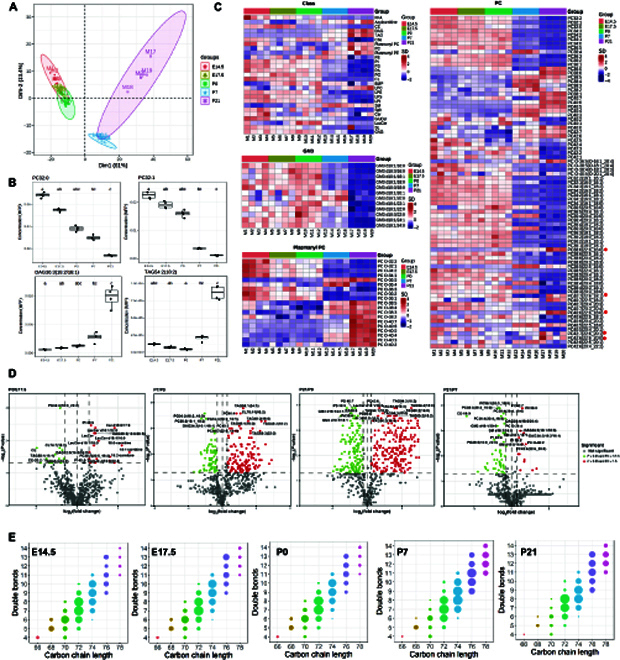
Developmental patterns of changes in cardiac mitochondrial lipidome. (A) Principal component analysis (PCA) of individual samples with bracketed numbers indicating the percentage of total variance explained by each component. (B) Box plots illustrate top altered mitochondrial lipids across cardiac development based on magnitude of *P* values. In all box plots, the median is indicated by the horizontal line and the first and third quartiles are represented by the box edges. The lower and upper whiskers extend from the hinges to the smallest and largest values, respectively, with individual samples indicated as dots. Changes were compared using ANOVA with post hoc Dunn’s test, and statistical significance was indicated using letter-based representation of all pairwise comparisons, such that 2 groups sharing a common letter are not significantly different at *P* < 0.05, *n* = 4 independent animals for each developmental stage. (C) Heatmaps illustrate overall changes in quantitated lipid classes and individual species belonging to the classes of GM3, plasmanyl PC, and PC of cardiac mitochondrial lipidome across development. *Z* scores of lipid levels expressed in MFP were plotted, *n* = 4 independent animals for each developmental stage; MFP: molar fractions normalized to total polar lipids. (D) Volcano plots display top lipids that were most significantly different in each pairwise comparison between mitochondrial lipidome at P0 relative to E17.5, P7 relative to P0, P21 relative to P0, and P21 relative to P7, based on magnitudes of *P* value and fold changes. Two-sided Dunn’s test was used for post hoc pairwise comparisons, *n* = 4 independent animals for each developmental stage. (E) Bubble plots illustrate compositional changes in mitochondrial CL profiles across cardiac development. CLs were classified according to total carbon atom numbers and number of double bonds. The bubble size represents the sum of all CL species (with different acyl compositions) that contained the defined total carbon atom numbers and total double bond numbers. *n* = 4 independent animals for each developmental stage. CL, cardiolipins.

To confirm mitochondrial-specific lipidomic changes across heart development, we isolated crude mitochondria fractions containing mitochondria and mitochondria-associated membranes (MAMs) from the respective developmental stages and carried out quantitative lipidomic analysis. Pure mitochondria devoid of MAM membranes were only isolated from P0 and P21 animals for confirmation of specific observed trends, as embryonic heart tissues from individual animals of prenatal stages were insufficient for isolation of pure mitochondria. A library of 587 lipids from 27 classes was quantitated (Fig. 4 and Fig. S3). We first used PCA to explore the global relationships among mitochondrial lipidomes of different developmental stages (Fig. 4A). In contrast to the whole-heart lipidome, mitochondrial lipidomes were clearly segregated based on metabolic status, with samples at postmetabolic transition (P7 and P21) clearly separated from the preceding developmental stages. The observations were consistent with the pivotal roles of mitochondria in determining cardiac metabolism. Top significantly altered mitochondrial lipids included short-chain, saturated/monounsaturated PCs (PC32:0 and PC32:1) that exhibited progressive decline across development and neutral lipids including DAG36:3(18:2/18:1) and TAG 54:2(18:2) that were increased sharply towards the end of cardiac metabolic transition (P7 to P21) (Fig. 4B). Heatmaps and volcano plots illustrate lipidomic changes in cardiac mitochondria across the developmental stages investigated (Fig. 4C and D). Numerous GM3s were markedly reduced at P21 relative to both P7 and P0, while several plasmanyl PCs were increased. Cardiac mitochondria at birth (P0) also exhibited appreciable elevations in several LacCer relative to prenatal E17.5 (Fig. 4C). In agreement with observations based on whole-heart lipidome, mitochondrial lipidomes also displayed increases in long-chain polyunsaturated PCs, particularly those containing DHAs, as development progresses (Fig. 4C, marked with an asterisk). Several TAGs were significantly increased after metabolic transition at P21 and P7 relative to P0 (Fig. 4D). The accretions in neutral lipid depots such as TAGs were specific to mitochondria and MAMs, as an overall reduction in TAGs was noted in whole-heart lipidome across development (Fig. 2, cluster 1, Fig. S1). Heatmaps summarizing the changes in mitochondrial lipid classes showed that P21 mitochondria possessed enhanced levels of neutral storage lipids, including TAG and DAG (Fig. 4C). Increases in specific mitochondrial TAGs at P21 were confirmed by analyses of pure mitochondria from P21 relative to P0 (Fig. S4A), which showed that several TAGs comprising long-chain (C54 to C58), polyunsaturated fatty acyls were increased in P21 mitochondria. Transmission electron microscopy (TEM) images revealed increasing contact points between lipid droplet (LD) and mitochondria as cardiac development progresses (Fig. S4B). While LDs mostly exist as stand-alone organelles in E17.5, they became increasingly associated with mitochondria across postnatal development (Fig. S4B). At P21, LD was closely flanked by 4 mitochondria on its periphery. The protein level of Perilipin 5 (Plin5), previously shown in neonatal cardiomyocytes to recruit mitochondria to LD surface via its C-terminal region [27], also increased in P21 crude mitochondria fractions compared to P7 (Fig. S4C).

### Integrated transcriptomics uncover candidate molecular drivers of membrane lipid remodeling

**Fig. 5.**
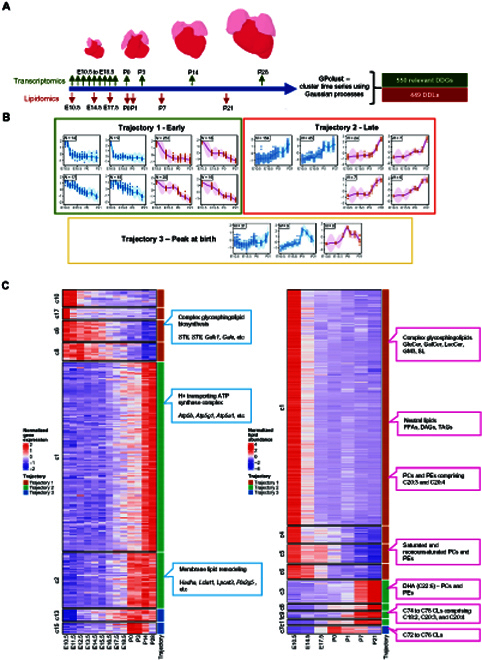
Integrated transcriptomics and lipidomics screen to study correlations between developmentally dynamic genes (DDGs) and developmentally dynamic lipids (DDLs) across cardiac development. (A) Diagram of sampling time points for the lipidomics study and the reference transcriptomics study. GPclust was used to model temporal changes of lipids and gene expression using the Gaussian processes model. (B) Curve and shaded region shows the predicted values and 95% confidence region of lipid/gene clusters from the GPclust model across heart development. Genes and lipids were selected using maSigPro, which showed significant temporal changes (FDR < 0.05). Genes and lipids showing that highly correlated variation patterns were assigned to 3 trajectories: 1, early; 2, late; and 3, peak at birth. Boxed number at the top left corner indicates the number of genes or lipids in each cluster. Blue line: gene cluster; red line: lipid cluster. (C) Heatmap plots of normalized gene expression and normalized lipid abundances (median of replicates) across heart development. The heatmap was split into blocks according to the cluster membership of individual gene or lipid (membership probability > 0.8). Trajectory assignment was indicated by color bars annotated on the right of each heatmap (red: Trajectory 1—early; green: Trajectory 2—late; blue: Trajectory 3—peak at birth). The detailed list of DDGs and DDLs classified under each developmental trajectory can be found in Tables S1 to S6.

Corroborating our observations based on whole-heart lipidome, mitochondria CL composition was remodeled toward species carrying longer (C76 to C78) and more unsaturated (number of C=C > 9) fatty acyls after metabolic transition (P7 to P21) (Fig. 4E), possibly to favor oxidative phosphorylation. In order to elucidate candidate drivers mediating acyl chain remodeling of CL and phospholipids during heart maturation, we mapped patterns of changes in developmentally dynamic genes (DDGs) (Fig. S5) published previously [17] with our observed DDLs (Fig. S6) using GPclust (see the Methods section) (Fig. 5 and Fig. S7). Changes in early DDGs corresponded with observations made based on early DDLs (Trajectory 1) (Fig. 5) and identified PE *N*-methyltransferase (*Pemt*) as a possible molecular driver underlying blunted conversions between cardiac PCs and PEs observed in postnatal stages. Other early DDGs included several genes mediating complex glycosphingolipid biosynthesis, including UDP-glucose ceramide glucosyltransferase (*Ugcg*) and ST8 alpha-*N*-acetyl-neuraminide alpha-2,8-sialytransferase (*St8sia1*), which were progressively reduced across cardiac development, concordant with the observed reductions in the levels of complex glycosphingolipids (GM3s, GluCer, and LacCer). Late DDGs (Trajectory 2) included CL remodeling genes such as *Hadha* and *Lclat1*, as well as various isoforms of phospholipases A (*Pla*) and lysophosphatidylcholine acyltransferase 3 (*Lpcat3*) key to phospholipid remodeling. Among the CL remodeling enzymes, only *Hadha* and *Lclat1* fell under Trajectory 2 in our analyses (Fig. 5). *Tafazzin* was previously reported as a late DDG in the developing mouse heart [17], which examined cardiac development up to P63. In our integrated GPclust analysis that monitored changes up to P28, however, the expression pattern of *Tafazzin* did not correlate strongly with changes in DDLs, which fell below the cutoff threshold to render classification as a late DDG under Trajectory 2. These observations suggest that *Tafazzin* might exert a lesser role compared to *Hadha* and *Lclat1* in modulating the CL profiles in early postnatal cardiac development. As for the remodeling of phospholipidome, we are interested to decipher molecular candidate(s) that mediate the accretion of DHA-phospholipids in cardiac membranes during cardiac metabolic transition, which should be represented by late DDGs that displayed progressively increasing expression patterns. Based on reported in vitro substrate specificity [28], candidate acyltransferases that incorporate DHAs into membrane PCs and PEs include Lpcat1, Lpcat2, Lpcat3, and Lpcat4, as well as membrane-bound *O*-acyltransferases MBOAT3 and MBOAT4. Among these candidates, only *Lpcat3* lay within Trajectory 2, while *Lpcat1* and *Lpcat4* fell under Trajectory 1. It is also noteworthy that 1-acylglycerol-3-phosphate *O*-acyltransferase 3 (Agpat3), known for its specific incorporation of DHAs into phosphatidic acids (PAs) [29,30], might offer an indirect route to the production of DHA-PCs and DHA-PEs from DHA-PAs via the cytidine diphosphate diacylglycerol (CDP-DAG) pathway. While *Agpat3* was also classified as a late DDG (Table S3), both PS synthase (*Ptdss2*) and *Pemt* were classified under Trajectory 1 as early DDGs (Table S1), making this alternative route of DHA-PC/PE production less likely. Thus, mapping transcriptome changes with lipidomic observations uncovered *Hadha*, *Lclat1*, and *Lpcat3* as possible mediators of CL remodeling and phospholipid remodeling, respectively, in the maturing mouse heart. Concomitant with the observed increases in cardiac mitochondrial DHA-phospholipids, several subunits of the H^+^ transporting ATP synthases (*Atp5b*, *Atp5g1*, and *Atp5a1*) on the IMM were among late DDGs of Trajectory 2 (Fig. 5). Also corresponding with our immunoblot results, *Plin5* was identified as a late DDG under Trajectory 2 (Table 3).

### Birth marks the structural and metabolic transition of mitochondria

**Fig. 6.**
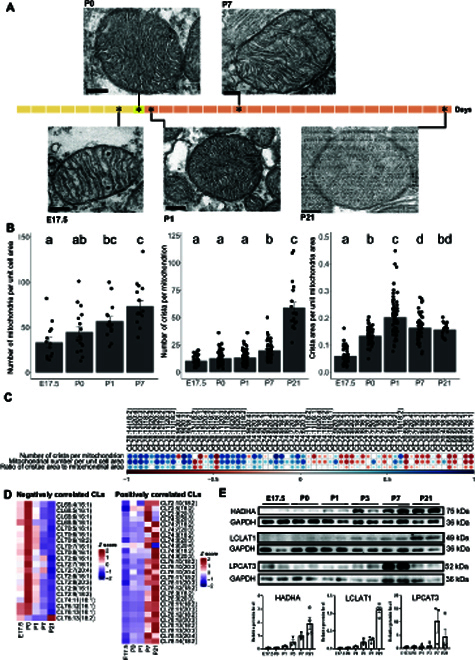
Cardiac mitochondria morphology in relation to membrane lipidome changes across development. (A) Transmission electron microscopy (TEM) images of mitochondria across heart development. Representative images from at least 2 independent experiments conducted in *n* = 3 (E17.5), *n* = 3 (P0), *n* = 3(P1), *n* = 3 (P7), and *n* = 2 (P21) animals were shown. Scale bars, 200 nm. (B) Changes in mitochondria-related indices including mitochondria number per unit cell area, number of crista per mitochondrion, and the ratio of cristae area to mitochondrial area across heart development. Changes were compared using ANOVA with Tukey’s HSD post hoc, and statistical significance was indicated using letter-based representation of all pairwise comparisons, such that 2 groups sharing a common letter are not significantly different at *P* < 0.05. Error bars were means + SEM. Mitochondria number per cell at individual stages were conducted in at least 3 randomly selected cells from *n* = 15 (E17.5), *n* = 15 (P0), *n* = 15 (P1), *n* = 15 (P7), and *n* = 10 (P21) TEM images. For measurements of crista number and crista proportional area, mitochondria with clear ultrastructures were randomly selected with the help of ImageJ software from *n* = 51 (E17.5), *n* = 48 (P0), *n* = 67 (P1), *n* = 48 (P7), and *n* = 20 (P21) TEM images. (C) Spearman correlation analysis evaluates correlations between individual species of CLs, arranged in descending order of total carbon atom numbers from the left to the right, with mitochondria maturation, as defined by the number of crista per mitochondrion (crista_num), number of mitochondria per unit cell area (mito_number), and crista area per unit mitochondria area (crista_area_per_mito_area) across 4 developmental stages including E17.5, P0, P1, and P7. Blue color indicates positive correlation, and red color indicates negative correlation; only statistically significant correlations at *P* < 0.05 were plotted. (D) Heatmaps illustrate developmental changes in the levels of heart CLs that displayed negative correlations (left panel) and positive correlations (right panel) with mitochondria maturation indices as defined by the mitochondria number per unit cell area, number of crista per mitochondrion, and crista area per unit mitochondria area. Only CLs with consistent correlations in one direction for all 3 indices were plotted. (E) Immunoblot analyses of protein levels of Hadha, Lclat1, and Lpcat3 across cardiac development. Images presented were representative of 2 experiments. Relative levels of individual proteins were normalized to that of GAPDH, and illustrated as bar plots on the lower panel. Error bars were means + SEM. *n* = 3, 3, 3, 3, 3, and 4 animals for E17.5, P0, P1, P3, P7, and P21, respectively. Hadha, hydratase subunit A/tri-functional protein alpha; Lclat1, lysocardiolipin acyltransferase 1; Lpcat3, lysophosphatidylcholine acyltransferase 3.

As mitochondria constitute the biological basis of metabolic transition in postnatal heart development, we used TEM to investigate and quantify changes in mitochondria morphology across development (Fig. 6). We observed a marked increase in the number and compactness of crista within mitochondria as development progresses. The number of mitochondria per unit cell area and the number of crista per mitochondrion increased progressively from E17.5 to P21, while the ratio of crista area to total mitochondria area peaked at P1 then dropped slightly afterwards (Fig. 6B). The peak in proportional crista area at P1 coincided with our observations on the TEM that mitochondria crista began to adopt a compact morphology with smaller intramembrane areas after P1. The increased compactness of mitochondria crista forms part of cardiac metabolic transition across the same period. As CLs are essential for the biogenesis and structural morphology of mitochondria crista, we next investigated the relationships between the abundances of individual CLs and mitochondrial maturation indices. Interestingly, we found that shorter CLs comprising predominantly fatty acyl C16:1 were negatively correlated with mitochondrial maturation indices, while longer CLs with more polyunsaturated fatty acyls (C18:2, C20:3, and C20:4) were positively correlated (Fig. 6D). The compositional abundances of negatively correlated CLs peaked at birth prior to metabolic transition, while those of positively correlated CLs were highest after metabolic transition (P7 to P21) (Fig. 6D). Finally, we verified by immunoblot analyses the protein steady-state levels of candidate molecular drivers that govern the remodeling of CL and DHA-phospholipids across cardiac development (Fig. 6E). Our results showed that in accordance with transcriptome profiles, protein levels of both Hadha and Lclat1 progressively increased across cardiac development. We did not conduct an immunoblot analysis of the Tafazzin enzyme for 2 reasons. First, Tafazzin did not qualify as a late DDG in our combinatorial GPclust analyses of lipidome and transcriptome data between E10.5 and P28. Second, reliable antibodies against mouse Tafazzin are not commercially available, as existing antibodies exhibit poor specificity and high cross-reactivity with multiple other proteins from the heart tissues [29,31,32]. Relative to earlier developmental stages, LPCAT3 was markedly increased at P7 and P21, during which mitochondria DHA-phospholipids accumulated (Fig. 4C).

## Discussion

Cardiac metabolic rewiring from glycolysis and lactate oxidation to β-oxidation of fatty acids commences shortly after birth at P0, and mitochondrial maturation completes at about 7 days after birth [33]. Our global analysis of whole-heart lipidome across developmental stages supports the dual roles of lipids in both structural maturation and metabolic transition. Decreases in cardiac acylcarnitines during development are metabolic adjustments to ensure normal cardiac function. Long-chain acylcarnitines were found to severely impair mitochondrial ATP production and compromise cardiac contractile function in zebrafish embryos [8]. Shortly following birth, the primary glucose transporter on sarcolemma switches from GLUT-1 to the insulin-responsive GLUT-4 isoform [34], and glucose uptake via GLUT-4 becomes the rate-limiting step in myocardial utilization of glucose. As GM3s function in glycosphingolipid-enriched microdomains to downregulate insulin signaling [35], postnatal reductions in GM3s may serve to enhance insulin sensitivity of cardiac tissues in rendering glucose uptake and utilization. Several enzymes mediating complex glycosphingolipid production were early DDGs; thus, postnatal reductions in complex glycosphingolipids such as GluCers and GM3s might have resulted from a redirected flow of Cer substrates away from complex glycosphingolipid production towards SM biosynthesis. Indeed, a temporal peak in cardiac SMs was observed at birth. Birth marks the transition from hyperplastic to hypertrophic growth of the heart [36]. Sphingosylphosphorylcholines, which carry identical chemical headgroups to SM but devoid of esterified fatty acyl chains, were reported to induce hypertrophic growth response in rat neonatal cardiomyocytes [37]. On the other hand, temporal increases in several LacCers in crude mitochondria fraction at birth may denote cellular adaptations in coping with increased energetic demands, as mitochondrial LacCers were shown to elevate glycolysis and oxidative phosphorylation [38]. Indeed, MAMs possess enzymatic activity that catalyzes the localized production of glycosphingolipids exclusively for the mitochondria [30].

Aside from mediating metabolic adaptations, changes in membrane lipidome also underlie critical structural adaptations with associated metabolic sequelae. We observed an overall increase in carbon atom number and degree of unsaturation in fatty acyls of major membrane lipid classes including PC, PE, and CL, concomitant with reducing ratio of Cho/PL and abated expression of *sqle* as the heart develops. Extensive increases in fatty acyl unsaturation accompanied by proportional reduction in Cho lead to elevated membrane fluidity, validated based on isolated sarcolemma using fluorescent DPH probes. In particular, the increasing unsaturation index of PCs and PEs was primarily attributed to increasing DHA incorporation, since C20:3 and C20:4 acyl substrates were increasingly transferred from PCs to PEs and then to CLs. The progressive reduction in *Pemt* expression across cardiac development might serve to prevent the flow of polyunsaturated fatty acid (PUFA) acyls from PEs back to PCs, given the substrate preference of *Pemt* for PEs carrying C18:2 and C20:4 acyls in their *sn-2* positions [39]. These observations support the findings from Oemer et al. [40] that the diversity of phospholipid acyl chains regulates the acyl chain modeling of mitochondrial CLs.

Acyl compositional changes in CLs and PEs are important to mitochondrial maturation and CL remodeling during heart development. First, both PCs and PEs denote sources of fatty acyls for transacylation during CL remodeling [24]. Our observation of an increasing positive correlation between CL and PE across postnatal cardiac development coincides with the known physical interaction between CL and PE on the IMM to form negatively curved monolayer facing inwards to the crista lumen [9]. Both CL and PE molecules adopt cone-shaped structures and denote non-bilayer phospholipids enriched in the inner layer of the IMM, while the outer layer adopts a positive curvature comprising mainly PCs (~80%) that faces the matrix. The interaction and enrichment of PE and CL on the IMM are essential for structural establishment of cristae morphology and the electron transport chain, since the loss of either PS decarboxylase that mediates IMM PE production [41] or CL remodeling activity [42] can lead to impaired oxidative phosphorylation. The assembly of electron transport chain components on the IMM results in protein crowding and packing stress, which can be relieved via PUFA-transacylation of CLs to increase membrane fluidity and negative curvature, leading to the formation of compact, stable lipid–protein complexes on the IMM [43]. Therefore, the coregulation between PEs and CLs and the postnatal remodeling of CL acyl chains are critical to the morphological maturation of tubular crista and organization of electron transport chain components on the IMM. Tubular cristae minimize luminal volume and increase the concentration gradient of internal solutes, particularly protons, thereby ensuring efficient ATP production via oxidative phosphorylation, which coincides with the metabolic skewing towards oxidative phosphorylation over glycolysis and lactate oxidation in postnatal cardiac maturation. Transcriptomics and immunoblot analyses identified *Hadha* and *Lclat1* as candidate players in the early phase of postnatal cardiac CL remodeling (P1 to P21) investigated herein. Corroborating our observations, *Hadha* was implicated in fatty acid β-oxidation and CL remodeling to ensure normal mitochondria calcium dynamics and repolarization kinetics critical in maintaining normal beat rate of human cardiomyocytes [14].

In the fully mature mammalian heart, CL 72:8(18:2) denotes the predominant species (i.e., >50% of total CL pool) [40,44]. Cardiac CL pool exhibits greater diversity in acyl compositions, however, during prenatal and early postnatal development [44], and a previous study showed that CL 72:8(18:2) forms the predominant species in the heart of mice only from 2 months onwards. In this study, we monitored cardiac development only up to 21 days after birth. In accordance with Chen et al. [44], we observed that CL72:7(18:2) denotes the most abundant CL in embryonic stages, and CL72:8(18:2) took over CL72:7(18:2) as the most endogenously abundant CL in the heart at P21 (Fig. 4E). In this study, during early cardiac metabolic transition that commences shortly after birth (P1 to P21), and prior to tetra-linoleoyl CL72:8(18:2) becoming the predominant species, we noted elevated levels of polyunsaturated C78-CLs concomitant with marked increases in steady-state protein levels of Hadha and Lclat1. Based on these results, we postulate that postnatal CL remodeling is biphasic, with Hadha and Lclat1 mediating an early phase of CL maturation to produce polyunsaturated C78-CLs, while Tafazzin enters later in the developmental picture to mediate the accumulation of tetra-linoleoyl CL72:8(18:2) in the fully mature heart, as demonstrated previously in Tafazzin-knockdown mice [29].

The effects of DHA accretion on cardiac phospholipidome are 2-fold, that is, in bringing about an overall increase in global lipid membrane fluidity, and in the unique structural properties that DHAs exert on mitochondrial membranes. Long-chain PUFAs, particularly DHAs, reduce the contraction rate of neonatal rat cardiomyocytes that is crucial for the prevention and cessation of lethal tachyarrhythmias (fibrillation) induced by calcium overload [45]. The protective, antiarrhythmic effects of DHAs are mediated via increasing sarcolemmal membrane fluidity [46]. Furthermore, increased rigidity of cardiac plasma membranes in cirrhotic cardiomyopathy results in impeded β-adrenergic receptor signaling and blunted cardiac contractility [47]. Therefore, establishment of high cardiac membrane fluidity is pivotal in maintaining normal, rhythmic contraction of cardiomyocytes as the heart matures. Dietary DHA supplementation in rats effectively enhances DHA esterification into cardiac mitochondrial phospholipids and specifically leads to delayed opening of mitochondrial permeability transition pores in both normal and hypertrophied hearts [48]. Manipulation of mitochondrial membrane DHAs via dietary supplementation also alters respiratory kinetics by promoting the activity of ATP synthase [49]. Indeed, several subunits of mitochondria ATP synthase were among the late DDGs along Trajectory 2 based on our integrated transcriptomics analysis. Our previous data are aligned with the membrane pacemaker theory of metabolism [9]. Esterified mitochondria DHAs may enhance the activity of H^+^ transporting ATP synthase on the IMM pivotal to the postnatal metabolic maturation of cardiac mitochondria. Similar positive effects of esterified DHAs were previously demonstrated on the molecular activity of sodium/potassium (Na^+^/K^+^) pumps on plasma membranes [50]. Molecular basis underlying the accretion of mitochondrial DHA-phospholipids remains to be elucidated, but our integrated analyses identified Lpcat3 as a plausible candidate. Nonetheless, the in vitro substrate specificity of Lpcat3 is not limited to DHAs alone, as it also effectively esterifies other unsaturated fatty acyls to lysophospholipid precursors [28]. It is also noteworthy that *Lpcat3* denotes the only acyltransferase categorized under the late DDGs of Trajectory 2 from our integrated omics analysis. In contrast, *Lpcat1* and *Lpcat4*, which also mediate the esterification of unsaturated PUFAs to lyso-phosphatidylcholines (LPCs) [28], were identified as early DDGs under Trajectory 1, which might underlie the reducing levels of C20:4/C20:3-PCs across cardiac development.

The accretion of neutral storage TAGs specific to mitochondria fractions from postnatal heart development denotes another interesting structural adaptation with metabolic implications. Such increases might be attributed to LDs that were copurified with mitochondria. Given the concomitant increases in both messenger RNA and protein levels of Plin5 revealed by transcriptomics and immunoblot analyses respectively, increasing formation of mitochondria–LD contacts over the course of cardiac development might lead to enhanced copurification of mitochondria-anchored LDs. Plin5 denotes an LD-associated protein that confers physical and metabolic bridging to the mitochondria [27]. Overexpression of *Plin5* enhances the formation of LD–mitochondria contacts, promoting mitochondria oxidation of fatty acyls and reducing cellular buildup of reactive oxygen species [51]. In oxidative tissues such as the brown adipose, LD and mitochondria form increasingly tight anchorage during tissue differentiation that remain unbroken by ultracentrifugation, which are thought to facilitate the channeling of fatty acids into mitochondria for β-oxidation to produce heat [52]. Likewise, the increasing mitochondria–LD contacts observed during cardiac development may be a structural adaptation to facilitate mitochondria oxidative phosphorylation, which preferentially utilizes medium-chain, saturated fatty acyls over polyunsaturated fatty acyls [53], thereby skewing the TAG profiles of purified mitochondria at P21 towards accumulation of long-chain, polyunsaturated TAG species.

This study has limitations. First, using our integrated approach of lipidomics and transcriptomics to identify molecular candidates governing global membrane remodeling across cardiac development falls short in uncovering regulation beyond transcriptional control. As changes documented at messenger RNA levels may not fully reflect protein steady states, and the mouse strains were different between Cardoso-Moreira et al. [17] and our study, we verified the candidate molecules identified from our multiomics screen with immunoblot analysis. While our screening approach revealed potential candidates, it is insufficient in itself to conclude that these candidates are molecular drivers of the reported lipidomic alterations during murine heart development. Extensive proteomics screening to reveal developmentally dynamic proteins (DDPs) denotes an interesting future pursuit to establish a more comprehensive screen of key enzymes governing cardiac development, which is beyond the scope of the current study. Validation via genetic knockdowns or knockouts is also necessary to establish causal relationships between the identified molecular candidates and lipidome alterations during cardiac development. Furthermore, while our correlation analyses indicated a flow of acyl substrates from PCs to PEs and finally to CLs that underlies CL remodeling in the maturing heart, the biological flux of fatty acyls can only be definitively concluded with metabolic flux tracing, for example, using isotope-labeled fatty acids. The lack of an untargeted analysis as a prescreen falls short in elucidating structurally novel lipids not previously reported in literature. Finally, our study timeline did not capture later developmental stages when cardiac CL maturation is complete, with tetra-linoleoyl CL constituting the predominant species of the CL pool. Despite these limitations, the datasets presented are a useful repository of dynamic lipidome alterations across cardiac development both within and outside the mitochondria.

To summarize, we present herein comprehensive whole-organ and mitochondria specific-lipidome atlases of the developing heart. Our lipidome analyses identified accretion of DHA-phospholipids and enhanced CL unsaturation as developmentally dynamic membrane lipid features governing structural and metabolic adaptations during cardiac maturation. We supplemented our lipidomic observations with systems integration of global transcriptomics data across prenatal and postnatal stages of murine heart development and uncovered *Lpcat3*, *Hadha*, and *Lclat1* as key molecular drivers behind mitochondria phospholipid and CL remodeling, respectively. Interestingly, both *Hadha* and *Lpcat3* were also late DDGs in human heart development, which suggested the translational significance of our findings. Our results support the membrane pacemaker theory of metabolism, which emphasizes the pivotal role of lipid membrane dynamics in determining cellular metabolism.

## Methods

### Collection of whole hearts across developmental time points

Protocols for all animal experiments were approved by the animal welfare committee of the Chinese Academy of Science, Institute of Genetics and Developmental Biology and Institute of Zoology. Whole hearts from the mouse strain (C57 BL/6N) were collected at 7 developmental time points along the prenatal and postnatal period (Fig. 1A), which included embryonic day 10.5 (E10.5) that marks the formation of basic heart chamber morphology, E14.5 during which atrial and interventricular septation completes, E17.5 at which definitive external prenatal configuration is attained; postnatal (P) periods included P0 at birth, P1 that marks the commencement of metabolic transition, P7 at which metabolic switch to reliance on fatty acid oxidation over lactate completes, and P21 at which weaning ends [36,54]. Whole hearts of each stage were dissected, washed with ice-cold phosphate-buffered saline 3 times, immediately snap-frozen with liquid nitrogen, and stored at −80 °C until further analyses.

### Mitochondria isolation from heart tissues

Isolation of mitochondria was carried out according to published protocol [55]. Harvested tissues were immediately washed 3 to 4 times with ice-cold starting buffer (SB; 225 mM mannitol, 75 mM sucrose, and 30 mM Tris-HCl [pH 7.4] at 4 °C) to remove traces of blood. Tissues were then cut into small pieces and washed again with SB to further remove blood traces, and homogenized in buffer HB-1 (225 mM mannitol, 75 mM sucrose, 0.5% BSA, 0.5 mM EGTA, and 30 mM Tris-HCl [pH 7.4] at 4 °C) using a prechilled Dounce homogenizer (KIMBLE) (10 strokes with A grinding pestle followed by 10 strokes with B grinding pestle). The homogenate was centrifuged at 740 *g* for 5 min at 4 °C. Clean supernatant was collected and centrifuged again at 740 *g* for 5 min at 4°C to further remove broken cells and nuclei. The clean supernatant was then centrifuged at 9,000 *g* for 10 min at 4 °C. The resultant supernatant containing microsomes and other cellular membranes was discarded, and the pellet containing mitochondria was resuspended in ice-cold HB-2 (225 mM mannitol, 75 mM sucrose, 0.5% BSA, and 30 mM Tris-HCl [pH 7.4] at 4 °C). The mitochondrial suspension was centrifuged at 10,000 *g* for 10 min at 4 °C. The mitochondrial pellet was resuspended in SB and centrifuged again at 10,000 *g* for 10 min at 4 °C. The final crude mitochondria pellet, which comprised mitochondria and MAMs, were resuspended in cold mitochondria resuspension buffer (MRB; 250 mM mannitol, 5 mM Hepes [pH 7.4], and 0.5 mM EGTA) and stored at −80 °C until further analysis. For further isolation of pure mitochondria with minimal MAM, crude mitochondria suspension was layered on top of Percoll medium (225 mM mannitol, 25 mM Hepes [pH 7.4], 1 mM EGTA, and 30% Percoll [vol/vol]) in the ultracentrifuge tube and topped up with MRB, then centrifuged at 95, 000 *g* for 30 min at 4 °C in a Beckman Coulter Optimal L-100 XP Ultracentrifuge with an SW40 rotor. A brownish dense band localized near the bottom of the tube containing purified mitochondria separated from MAM was transferred to a fresh tube. The mitochondrial fraction was washed twice with MRB (6,300 *g* for 10 min at 4 °C). Crude mitochondrial preparations were used for lipidome analysis of 5 developmental time points (E14.5, E17.5, P0, P3, and P7) spanning across prenatal and postnatal development, as embryonic heart tissues harvested from individual animals were insufficient for isolating pure mitochondria devoid of MAM. Analyses of crude mitochondria with MAM also offer an added advantage in terms of interrogating interorganelle metabolic crosstalk during heart development. To confirm changes in lipidomes specific to mitochondria during postnatal metabolic rewiring, we further collected pure mitochondria from the heart tissues of P0 and P21 animals and performed lipidomic analyses. Purities of crude mitochondria and pure mitochondria samples were assessed via immunoblot analysis (Fig. S9).

### Lipid extraction

Lipids were extracted from heart tissues using the Bligh and Dyer’s protocol as previously described [56]. Briefly, 900 μl of chloroform:methanol (1:2) containing 10% deionized H_2_O was added to each sample, which was then homogenized on an automated bead ruptor (OMNI, Seattle, WA, USA) with an optimized program (5 m/s; 8 s; 2 cycles; pause 5 s). Following homogenization, samples were incubated at 1,500 rpm at 4 °C for 1 h. At the end of incubation, 400 μl of deionized H_2_O and 300 μl of chloroform were added to induce phase separation. The lower organic phase was transferred to a fresh tube. A second round of extraction was performed via the addition of 500 μl of chloroform. The organic extracts from both rounds of extraction were pooled and dried using SpeedVac under OH mode and channeled for lipidomic analyses.

### Mass spectrometric analyses

Whole lipidome was analyzed by targeted multiple-reaction monitoring (MRM) based on 5 separate injections catering to specific classes of lipids (Table S7) and reported according to guidelines [57]. Neutral lipids including TAGs and DAGs were quantitated using a modified version of reverse-phase HPLC/MRM on an Agilent 1260 HPLC coupled to SCIEX QTRAP 5500 under electrospray ionization positive mode [58]. Separation of neutral lipids was achieved on a Phenomenex Kinetex C18 2.6-μm column (internal diameter 4.6 × 100 mm) using an isocratic mobile phase containing chloroform:methanol:0.1 M ammonium acetate (100:100:4 v/v/v) at a flow rate of 300 μl for 10 min. Mass spectrometer (MS) source parameters were as follows: CUR 10, TEM 350 °C, GS1 35, and GS2 35. Levels of short-, medium-, and long-chain TAGs were calculated by referencing to spiked internal standards of TAG(14:0)_3_-d_5_, TAG(16:0)_3_-d_5_, and TAG(18:0)_3_-d_5_ from CDN isotopes, respectively. DAGs were quantified using DAG(16:0/16:0)-d_5_ and DAG(18:1/18:1)-d_5_ from Avanti Polar Lipids as internal standards for saturated and unsaturated species, respectively. Free Cho and total cholesteryl esters were quantified using atmospheric pressure chemical ionization in the positive mode on an Agilent 1260 HPLC coupled to SCIEX QTRAP 5500 as elaborated previously [59]. Cho and its esters were separated on an Agilent Eclipse XDB C18 5-μm column (internal diameter 150 × 4.6 mm) using an isocratic mobile phase comprising chloroform:methanol (1:1 v/v) at a flow rate of 700 μl/min. MS source parameters were as follows CUR 20, TEM 500 °C, GS1 45, and GS2 35. Phospholipids and SPLs from mitochondrial samples were analyzed using electrospray ionization under both positive and negative modes on a Jasper HPLC coupled to Sciex 4500 MD, while that from whole hearts were analyzed on an Exion UPLC coupled to Sciex QTRAP 6500 Plus. A total of one injection in the negative mode (PE, PG, PI, PA, PS, BMP, CL, GM3, SL, FFA, LPE, LPI, LPA, LPS, and PC with fatty acyl-specific transitions) and 2 injections in the positive mode (PC, LPC, SM, Cer, GluCer, LacCer, and Sph) were made. In order to ensure that the intensities of all detected lipids fall within the linearity range of the mass spectrometer for quantification, 2 separate injections of samples at different dilutions were made in the positive modes to cater for the stark differences in endogenous abundances between endogenously abundant PCs and SMs relative to the remaining SPLs. MS source parameters for 45 MD were as follows: CUR 20, TEM 450 °C, GS1 45, and GS2 45. MS source parameters for QTRAP 6500 Plus were as follows: CUR 20, TEM 400 °C, GS1 20, and GS2 20. Phospholipids and SPLs were separated on a TUP HB Silica 3-μm column (150 × 3 mm) using chloroform:methanol: aqueous ammonia (895:100:5 v/v/v) as mobile phase A and chloroform:methanol:water:aqueous ammonia (270:650:70:10 v/v/v/v) as mobile phase B. The gradient started with 2% B for the first 2 min and underwent 3 incremental increases in %B to 35% at the third minute, 55% at the fifth minute, and 85% B at the sixth minute. The gradient was maintained at 85% B for 1 min, and further increased to 100% B within 0.2 min. The gradient was maintained at 100% B over 3.8 min then decreased back to 2% B over 0.5 min. The column was equilibrated at 2% B for 4.5 min before the next injection. Individual lipids were quantitated relative to their respective internal standards, which included d_9_-PC32:0(16:0/16:0), DMPC, d_9_-PC36:1p(18:0p/18:1), d_7_-PE33:1(15:0/18:1), PE14:0/14:0, d_9_-PE36:1p(18:0p/18:1), d_31_-PS(16:0/18:1), d_7_-PG33:1(15:0/18:1), PG14:0/14:0, d_7_-PI33:1(15:0/18:1), d_7_PA33:1(15:0/18:1), PA 17:0/17:0, BMP 14:0/14:0, d_5_-CL 72:8(18:2)_4_, d_8_-SM d18:1/18:1, SM d18:1/12:0, Cer d18:1/d_7_-15:0, Cer d18:1/17:0, GluCer d18:1/8:0, d_3_-LacCer d18:1/16:0, GM3 d18:1/18:0-d_3_, Gb3-d18:1/17:0, SL-d18:1/17:0, d_7_-LPC 18:1, LPC C17:0, d_7_-LPE 18:1, LPE C17:0, LPA-C17:0, LPI-C17:1, LPS-C17:1, LPG-C17:1, S1P-d17:1, and Sph-d17:1 obtained from Avanti Polar Lipids; d_3_-16:0-carnitine from Cambridge Isotope Laboratories; diC8-PI from Echelon Biosciences; and d_31_-FFA-16:0 from Sigma-Aldrich and d_8_-FFA-20:4 from Cayman Chemicals. Quality control (QC) samples, which were pooled from biological samples, were inserted every 10 actual biological samples across the MS run. The polar aqueous phase from the extraction was used for analysis of acyl-CoAs on a system comprising Thermo Fisher DGLC U3000 coupled with QTRAP 6500 Plus from Sciex, as previously described in the original method paper [23]. 19:0-CoA from Avanti Polar Lipids was used for the relative quantitation of acyl-CoAs.

### Transmission electron microscopy

Heart tissues were cut into 1 mm^3^ on ice, fixed in 2.5% glutaraldehyde, washed, postfixed in 1% osmic acid, and electron-stained in anhydrous acetone with 0.01% uranyl acetate followed by gradual anhydrous acetone, then infiltrated with Embed-812 resin as follows: resin/acetone (v/v) 1:1 for 2 h, 2:1 for 2 h, 3:1 for 2 h, and twice with 100% resin for 12 h each, and finally embedded at 65°C for 72 h. The fixed samples were cut into 70-nm sections with a microtome EM UC7 (Leica Biosystems). Sections were observed using a JEM-1400 (JEOL) operating at 80 kV, as described previously [60]. Measurements of mitochondrial suborganellar structures were performed using ImageJ. Mitochondria number per cell at individual stages were determined in at least 3 randomly selected cells from *n* = 15 (E17.5), *n* = 15 (P0), *n* = 15 (P1), *n* = 15 (P7), and *n* = 10 (P21) TEM images. For measurements of crista number and crista proportional area, mitochondria with clear ultrastructures were randomly selected with the help of ImageJ software from *n* = 51 (E17.5), *n* = 48 (P0), *n* = 67 (P1), *n* = 48 (P7), and *n* = 20 (P21) TEM images.

### Sarcolemma isolation from heart tissues

Sarcolemma isolation from heart tissues was performed as previously described [61]. Whole heart was rapidly excised and washed with homogenizing medium (250 mM sucrose and 10 mM Tris-HCl, pH 7.6, at 20 °C). Tissues were then homogenized with a prechilled Dounce homogenizer (KIMBLE) (10 strokes with A grinding pestle followed by 10 strokes with B grinding pestle), and the homogenate was filtered through a 100-μm cell strainer. The filtered homogenate was topped up with 3 M KCl and 200 mM sodium pyrophosphate to make final concentrations of 300 mM KCl and 25 mM sodium pyrophosphate, respectively. The homogenate was then immediately centrifuged for 45 min at 177,000 *g*. The supernatant was discarded and the pellet was then resuspended in 0.125% DNase I homogenizing medium and incubated for 90 min at 37 °C. The resuspension was homogenized with 10 strokes using B grinding pestle and then centrifuged for 8 min at 200 *g*. The supernatant was carefully removed and centrifuged for 45 min at 177,000 *g* again. The resulting pellet was resuspended in 1.4 ml of 45% sucrose, and a discontinuous sucrose gradient containing 1.9 ml of 20% and 2.4 ml each of 27%, 30%, 32%, and 34% sucrose (w/w) was layered on top of the resuspension. The gradient was centrifuged for 16 h at 122,000 *g*. Sarcolemma membrane fractions were collected primarily in the vicinities of fractions 2 and 3 (~27% sucrose).

### Membrane anisotropy measurement

Lipids were extracted from aliquots of the isolated sarcolemma membranes to quantitate the content of PCs. Membrane anisotropy measurements were performed using the fluorescent dye DPH as previously described [56]. Briefly, 0.44805 nmol equivalent of PCs from sarcolemma membranes, quantitated using LC-MRM with d_9_-PC32:0 (16:0/16:0) as an internal standard, was resuspended in 650 μl of 1 mM Tris-HCl buffer containing protease inhibitors at pH 7.4. Labeling of sarcolemma membrane was performed at a molecular ratio of 1 probe:360 PCs at 30 min under room temperature with gentle agitation in the dark. The labeled samples were measured at 37 °C with vertically polarized light in a Hitachi F-7000 fluorescence spectrophotometer. Emission intensities were measured parallel (*I*vv) and perpendicular (*I*vh) to the vertical plane of polarization. To eliminate the effect of instrument polarization response, grating correction factor (*G*) was taken into consideration, calculated by exciting the samples with horizontally polarized light and measuring the parallel and perpendicular components, where *G* = *I*hv/*I*hh. Anisotropy (*γ*) was calculated according to the formula *γ* = (*I*vv − *GI*vh)/(*I*vv + 2*GI*vh).

### Immunoblot analysis

Proteins were extracted using RIPA lysis buffer with protease inhibitor cocktail (Sigma-Aldrich). The Pierce BCA protein assay kit (Thermo Fisher Scientific) was used to determine protein content. Ten micrograms of mitochondrial proteins was subjected to 12.5% sodium dodecyl sulfate–polyacrylamide gel electrophoresis (SDS-PAGE) and blotted onto nitrocellulose membranes (Pall). Primary antibodies included the following: anti-Bip (Beyotime, AB310, 1:1,000), anti-Caveolin-1 (CST, 3238S, 1:1,000), anti-Cytochrome C (Abcam, ab110325, 1:2,000), anti-Cox IV (Abcam, Ab16056,1:2,000), anti-Lamin B1 (CST, #12586, 1:2,000), anti-Plin5 (Novus, NB110-60509, 1:500), anti-Hadha (Pierce, PA529813, 1:500), anti-Lpcat3 (Abcam, ab239585, 1:500), anti-Lclat1 (Bioss, bs-18190R, 1:500), anti-PTDSS1 (Abcam, ab157222, 1:1,000), and anti-GAPDH (CUSABIO, CSB-MA00007, 1:500). Secondary antibodies included the following: goat anti-rabbit HRP (ZSGB-BIO,ZB-2305, 1:5,000) and goat anti-mouse HRP (ZSGB-BIO,ZB-2301, 1:5,000).

### Statistical analysis

All statistical analyses were performed using R 4.1.0. Internal standard calibration was used for relative quantification of lipids [62]. An aliquot of the lipid extracts was mixed with known concentrations of an internal standard cocktail in a 1:1 (v/v) ratio, and injected into the mass spectrometer. Amounts of individual lipids were quantitated using this equation:

$$\begin{array}{c} \text{Amount of lipid}\;X\;\text{in μmol}=\\ \;\;\;\frac{\text{Peak area of lipid}\;X*\text{Concentration of internal standard in μmol}/\mathrm{ml}}{\text{Peak area of selected internal standard}}\\ \;\;\;*\text{Sample resuspension volume}\;\left(\mathrm{ml}\right) \end{array}$$

The amounts of individual lipids expressed in micromoles were normalized to the sum total of all phospholipids and SPLs detected in the sample (also expressed in micromoles). The resultant molar fractions normalized to total polar lipids (i.e., MFP) were used for subsequent statistical analyses. MFPs were used for hypothesis tests and correlation analyses, and transformed to *Z* scores before PCA and clustering analyses.

#### Elucidation of DDLs

Based on developmental time-course lipidomic data, we identified DDL species using maSigPro, an R package for identifying significantly differential expression profiles in time-course data. A polynomial linear regression (degree = 3) model was fitted on the normalized (centered and scaled) lipid concentration values. Lipids were considered as DDLs when corrected significance level (Benjamini–Hochberg method) is less than 0.05, and goodness of fit (*R*^2^) is higher than 0.7 in the stepwise-selected (backward method) regression model.

#### Integration of developmental trajectories based on lipidome and transcriptome profiles

Published gene expression data of developing mouse heart [17] was retrieved from https://www.ebi.ac.uk/arrayexpress/experiments/E-MTAB-6798/. GPclust was used to cluster time series using Gaussian processes on the 550 DDGs manually selected based on their curated functions with respect to lipid metabolism, mitochondrial metabolism, and peroxisome metabolism and 448 DDLs. We then used Matern52 as the underlying kernel and with variance equal to 1 and length scale equal to 2 for the mean function of each cluster. Matern52 kernel (variance = 0.7, length scale = 2) together with white kernel (variance = 0.1) were used for evaluating the deviation of individual gene/lipid from the cluster mean. The average expression profile of each cluster was predicted using the GPclust model for time points E10.5, E11.5, E12.5, E13.5, E14.5, E15.5, E16.5, E17.5, E18.5, P0, P1, P3, P7, P14, and P21, which were assigned to integer values 1 to 15. A gene or lipid was considered to be a true member of the cluster if the probability is higher than 0.8 for that cluster. Aligned predicted average values were compared between every cluster of lipid and gene profiles using Spearman correlation. Interomics clusters with strong positive correlation (FDR < 0.01) and highly similar trajectory were identified for further investigation.

#### Other analyses

The distributions of carbon atom numbers and double bond numbers across major lipid classes in whole hearts at Day 21 were illustrated using radar diagrams drawn with ggplot2. PCA and cluster dendogram analysis were conducted on log-transformed and standardized data using FactoMineR to first explore the global relationships among the samples. Fuzzy c-means clustering was performed on log-transformed, standardized data to identify distinct temporal patterns of lipid changes across development. Temporal changes in the abundances of major lipid classes were compared using ANOVA with Tukey’s HSD post hoc and illustrated using bar plots with letter-based representation of all pairwise comparisons, such that 2 groups sharing a common letter are not significantly different at *P* < 0.05. We next assessed the fluidity changes in myocardial cell membranes across development using several indicators, including fraction of total PLs, fraction of total Cho, ratio of Cho/PL, and fraction of SPLs using ANOVA with Tukey’s HSD post hoc, and illustrated using bar plots with letter-based representation of all-pairwise comparisons, such that 2 groups sharing a common letter are not significantly different at *P* < 0.05. Pie charts illustrating changes in the proportion of major SPL classes across the 7 selected time points of heart organogenesis were drawn using the plotly package. Correlation between mitochondrial indices including the number of crista per mitochondrion, the number of mitochondria per unit cell area, and the levels of individual CLs was analyzed using the corrplot package in R. Blue shade indicates positive correlation, while red shade indicates negative correlation. Size of circles indicates magnitude of correlation; only correlations with *P* < 0.05 were plotted. Bubble plots constructed using ggplot2 (v3.3.3) illustrate the relative abundances of cardiolipin species grouped by total number of carbon atoms and double bonds in the acyl chains across each developmental stage, where the bubble size represents the sum of all cardiolipin species (with different acyl compositions) that contained the defined total carbon atom numbers and total double bond numbers.

## Data Availability

Source datasets are available on Mendeley Data at http://dx.doi.org/10.17632/56hkkb5kdg.1.

## Supplementary Materials

Supplementary 1 Fig. S1. Whole-heart lipidomes across cardiac development. Fig. S2. Distinct patterns of lipid changes across heart development. Fig. S3. Mitochondria lipidomes across cardiac development. Fig. S4. Enhanced mitochondria–lipid droplet contacts in postnatal cardiac development mediated by Plin-5. Fig. S5. Temporal changes of gene expression in the clusters determined by GPclust. Fig. S6. Temporal changes of lipid profiles in the clusters determined by GPclust. Fig. S7. Correlation of temporal changes between the clusters determined by GPclust of gene expression and lipids. Fig. S8. Relative gene expression levels of *Hadha* and *Lpcat3* in human heart development. Fig. S9. Immunoblot assessment on the purity of crude mitochondria and pure mitochondria fractions isolated from P21 heart tissues. Fig. S10. Quality control across MS runs.

Supplementary 2 Table S1. DDGs in Trajectory 1 (early). Table S2. DDLs in Trajectory 1 (early). Table S3. DDGs in Trajectory 2 (late). Table S4. DDLs in Trajectory 2 (late). Table S5. DDGs in Trajectory 3 (peak at birth). Table S6. DDLs in Trajectory 3 (peak at birth). Table S7. Lipid identification approach. Table S8. Method LOD and LLOQ. Table S9. Table of MRM transitions for reported lipids.
