# Erratum to “Lipidome Atlas of the Developing Heart Uncovers Dynamic Membrane Lipid Attributes Underlying Cardiac Structural and Metabolic Maturation”

**DOI:** 10.34133/research.0185

**Published:** 2023-06-26

**Authors:** Huan Miao, Bowen Li, Zehua Wang, Jinming Mu, Yanlin Tian, Binhua Jiang, Shaohua Zhang, Xia Gong, Guanghou Shui, Sin Man Lam

**Affiliations:** ^1^ State Key Laboratory of Molecular Developmental Biology, Institute of Genetics and Developmental Biology, Chinese Academy of Sciences, Beijing 100101, China.; ^2^ University of Chinese Academy of Sciences, Beijing 100049, China.; ^3^ LipidALL Technologies Company Limited, Changzhou 213022, Jiangsu Province, China.

## Main Text

In the Research Article “Lipidome Atlas of the Developing Heart Uncovers Dynamic Membrane Lipid Attributes Underlying Cardiac Structural and Metabolic Maturation” [1], there was an error in Fig. 2B. The authors mistakenly repeated the chord diagram designated for P7 with that from P1. The figure amendment did not affect the in-text discussion of the figure, which was focused on changes between P0 and P21, and did not alter the conclusion. Figure 2B has now been corrected in the PDF and HTML (full text).

**Fig. 2.**
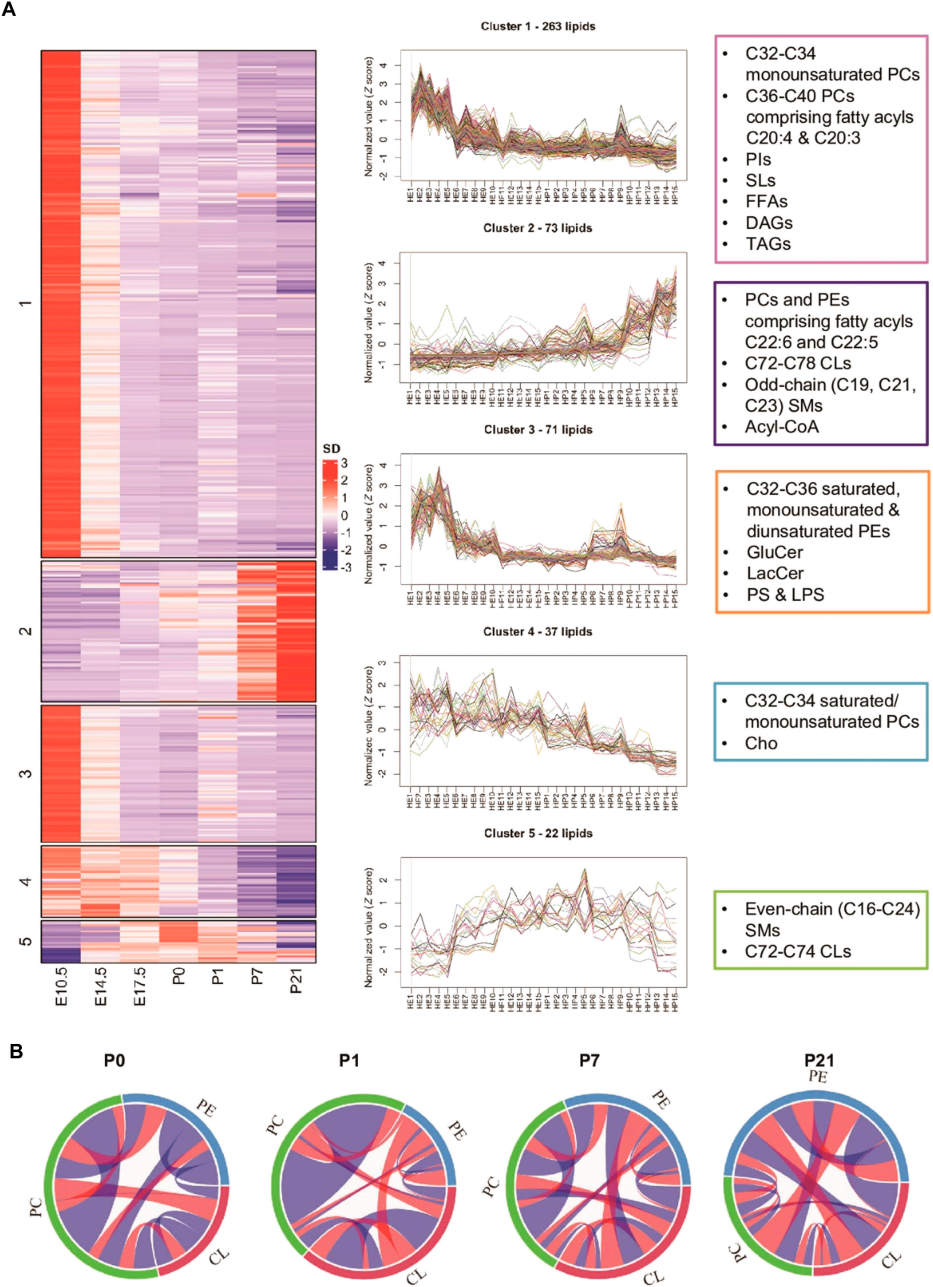
maSigPro identified 5 clusters of developmentally dynamic lipids (DDLs) in the heart. (A) Lipid species were partitioned into 5 clusters by hierarchical clustering algorithm according to their variation patterns. The left panel shows row-normalized abundance (median of replicates) of lipid species with significant temporal changes across heart development. The middle panel shows changes in individual lipid species across samples ordered by developmental time points. Five clusters of DDLs were identified across prenatal and postnatal stages of heart development. Cluster 1 (263 lipids) and cluster 3 (71 lipids) denote early DDLs (highest levels in early developmental stages), cluster 2 (73 lipids) represents late DDLs (highest levels in late developmental stages), cluster 4 (37 lipids) comprises DDLs that peaked shortly prior to birth, and cluster 5 (22 lipids) consists of DDLs that peak at birth and then decrease. Representative lipids of each cluster are boxed and illustrated on the right panel. (B) Chord diagrams illustrate changes in lipid correlations among classes of CL, PC, and PE across postnatal heart development. Correlations between lipids were calculated using Spearman’s correlation analysis in each postnatal developmental stage. Band width indicates the number of significant correlations, and color indicates direction of correlation. *P* value cutoff was set at *P* < 0.05. Blue shade indicates positive correlations, while red shade indicates negative correlation between 2 connecting lipids.
