# Supplementary material for: Lipidome Atlas of the Developing Heart Uncovers Dynamic Membrane Lipid Attributes Underlying Cardiac Structural and Metabolic Maturation
Source: Research (Wash D C). 2022 Dec 19;2022:0006. doi: 10.34133/research.0006 (PMC11407523; doi:10.34133/research.0006)

SUPPLEMENTAL FIGURE 1

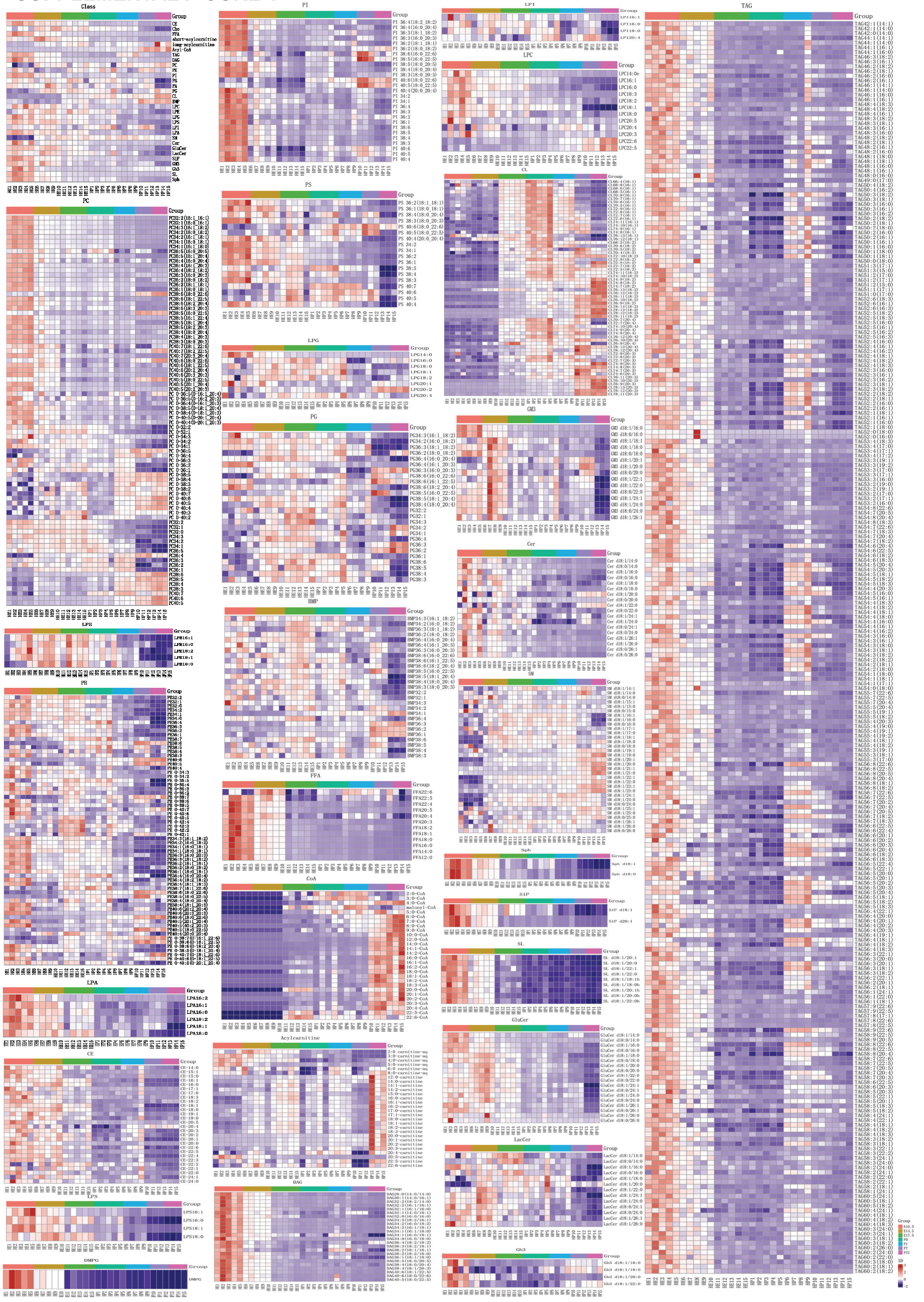

SUPPLEMENTAL FIGURE 2

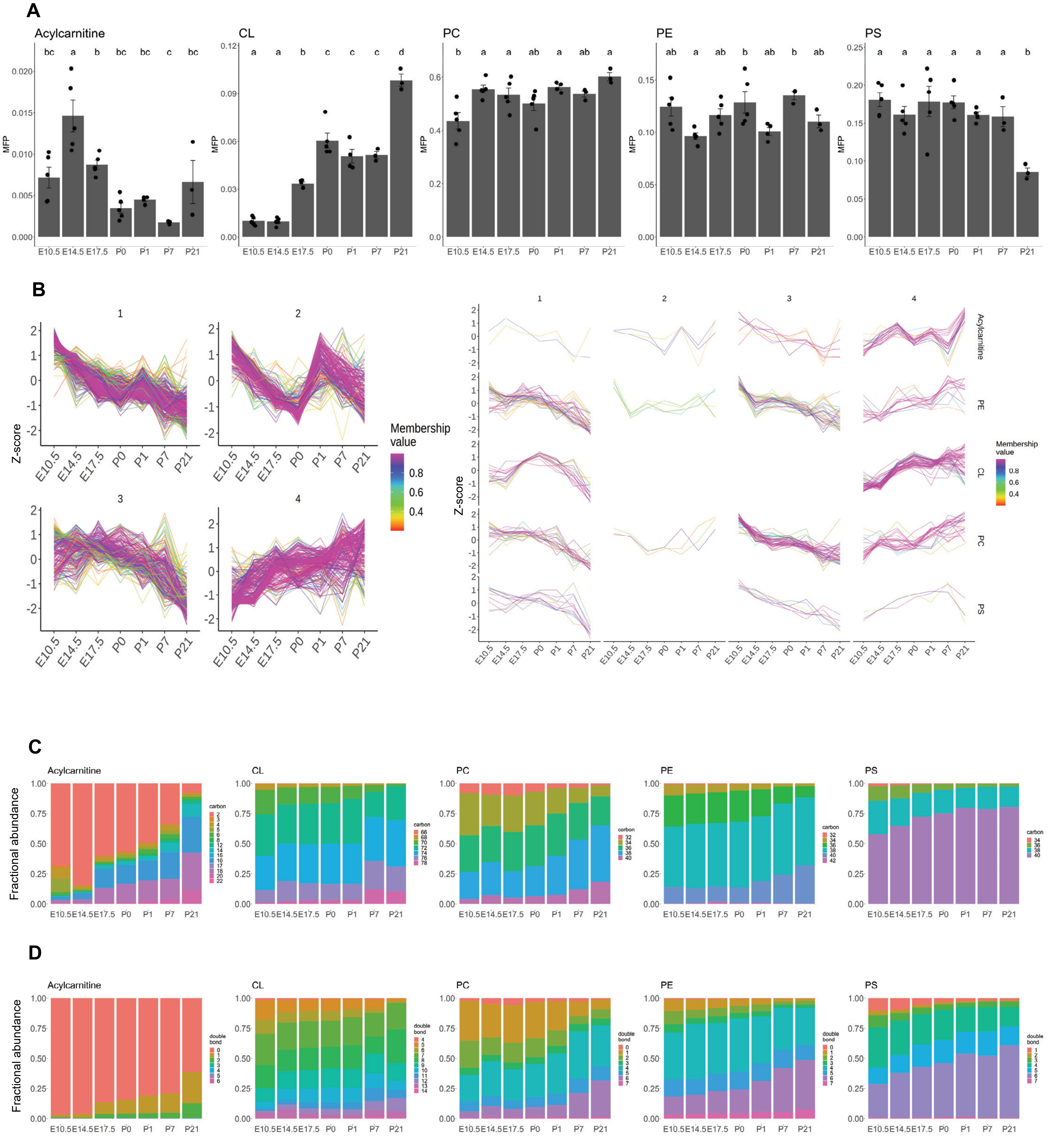

SUPPLEMENTAL FIGURE 3

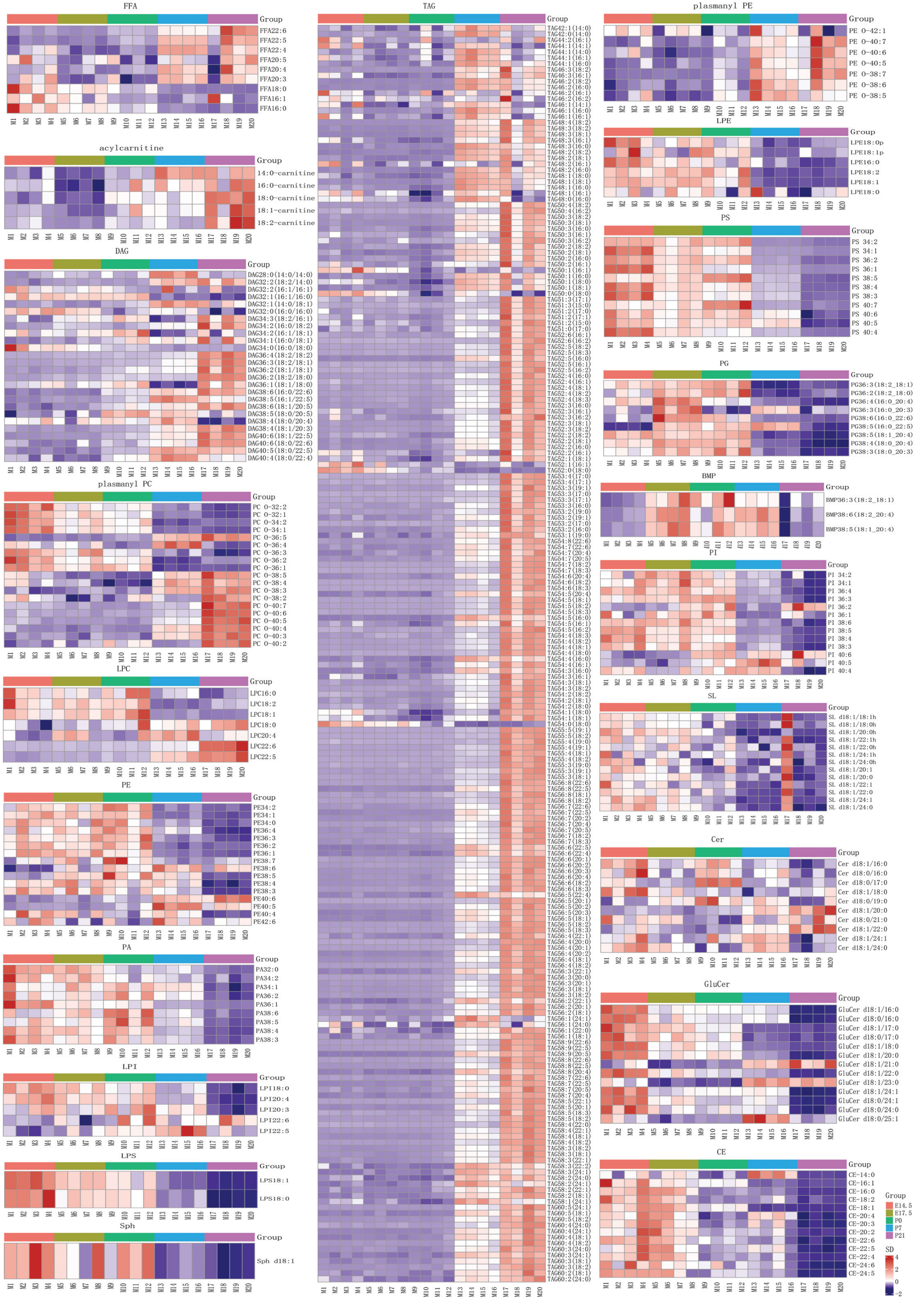

SUPPLEMENTAL FIGURE 4

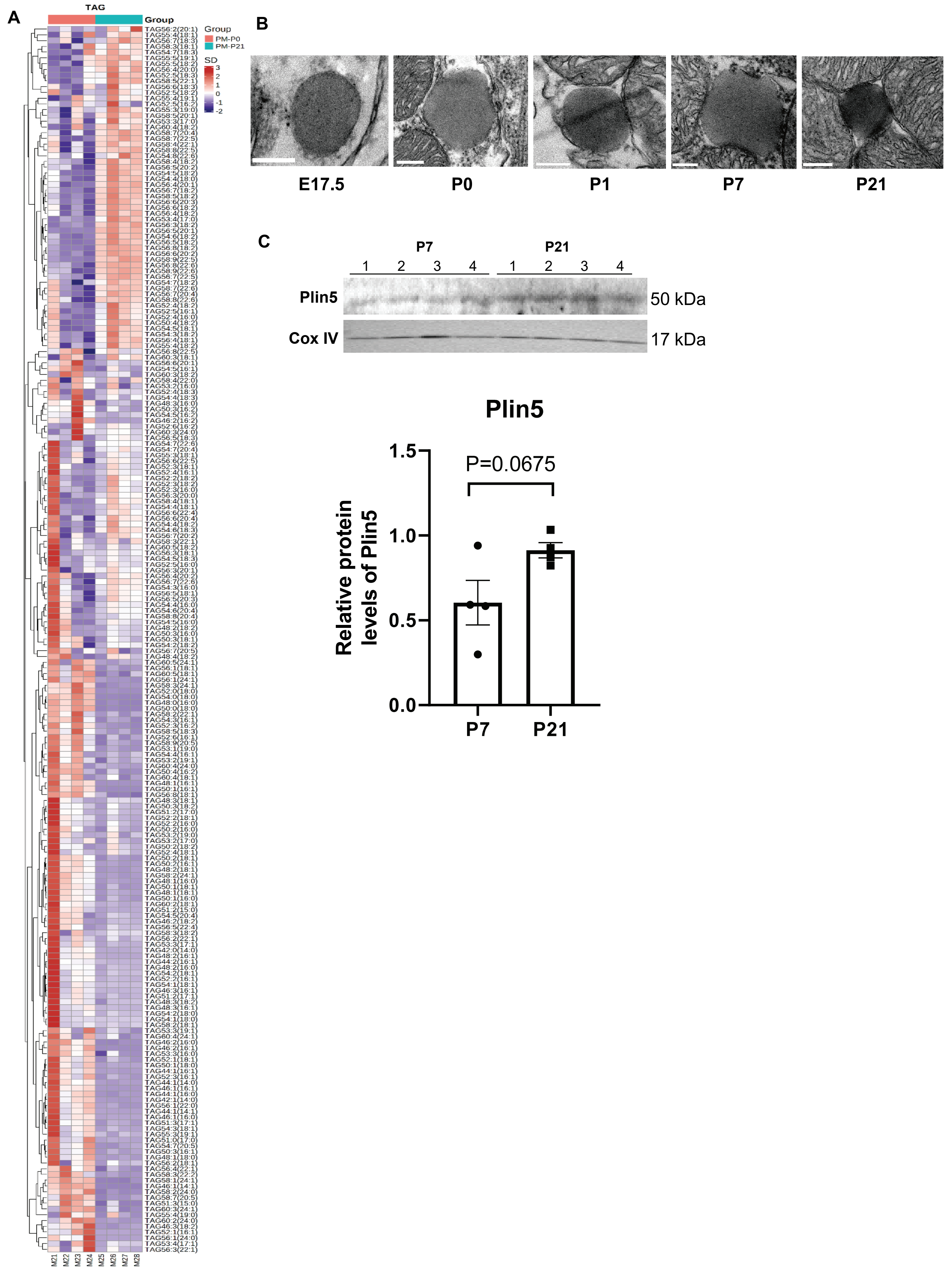

SUPPLEMENTAL FIGURE 5

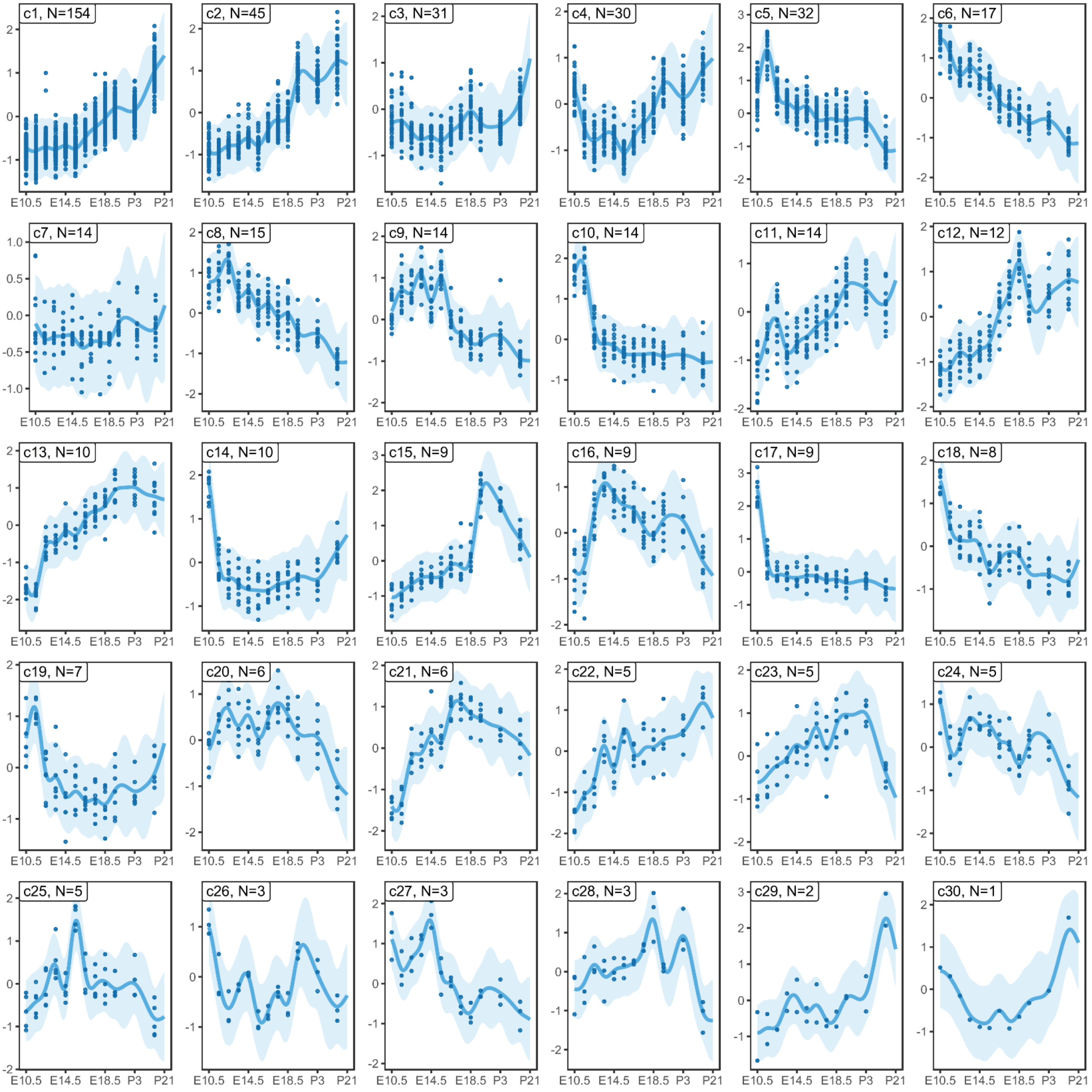

SUPPLEMENTAL FIGURE 6

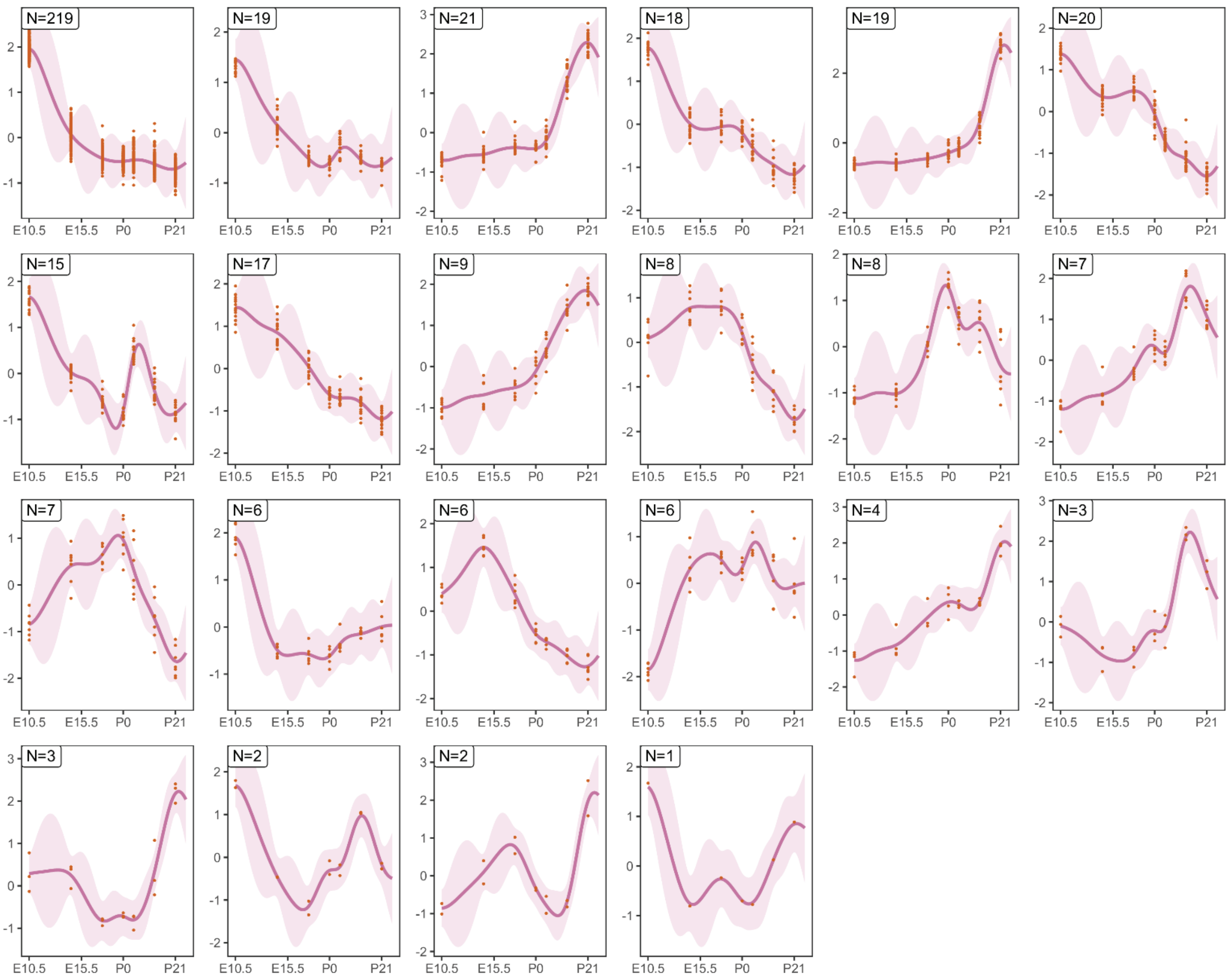

SUPPLEMENTAL FIGURE 7

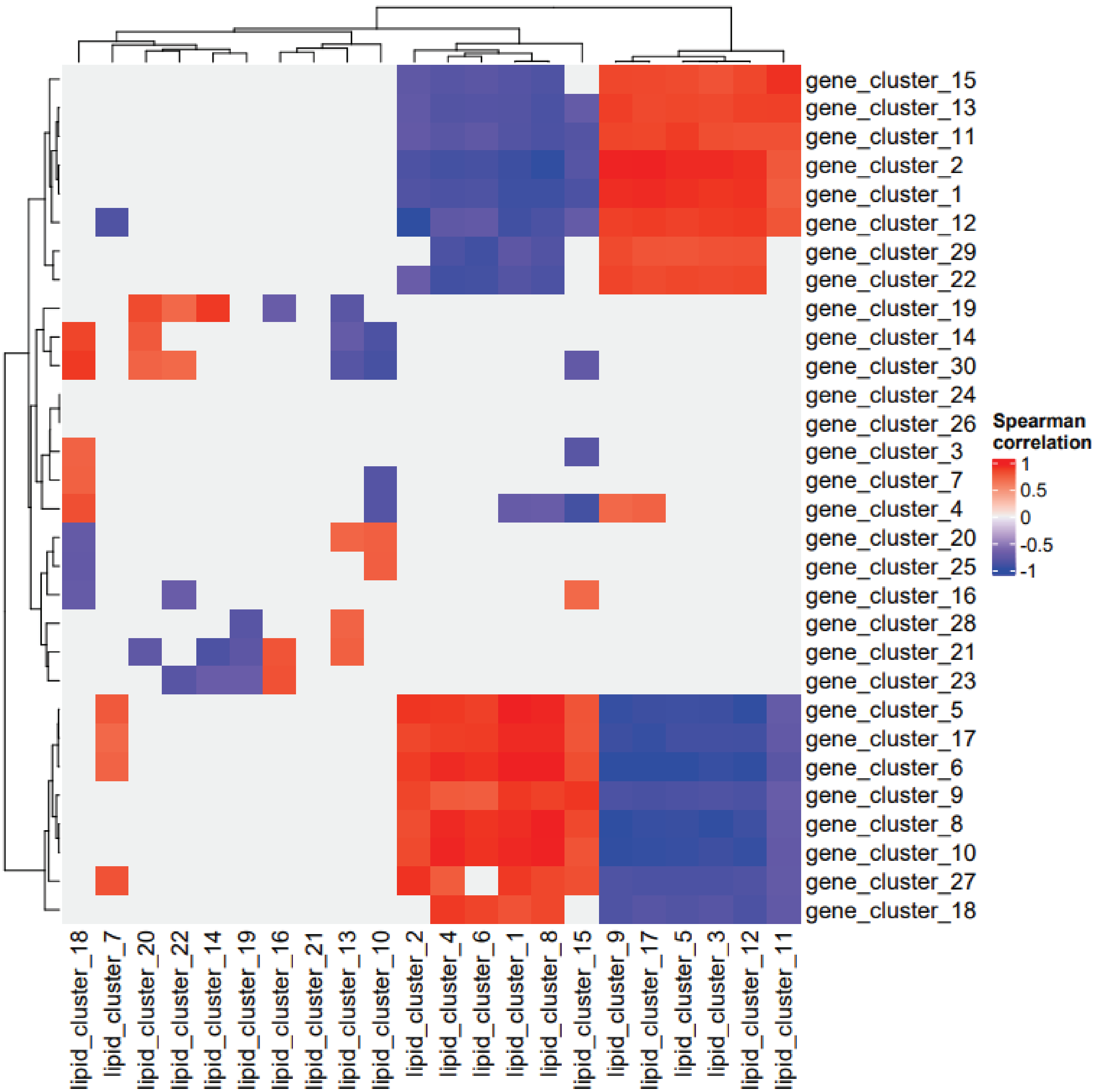

SUPPLEMENTAL FIGURE 8

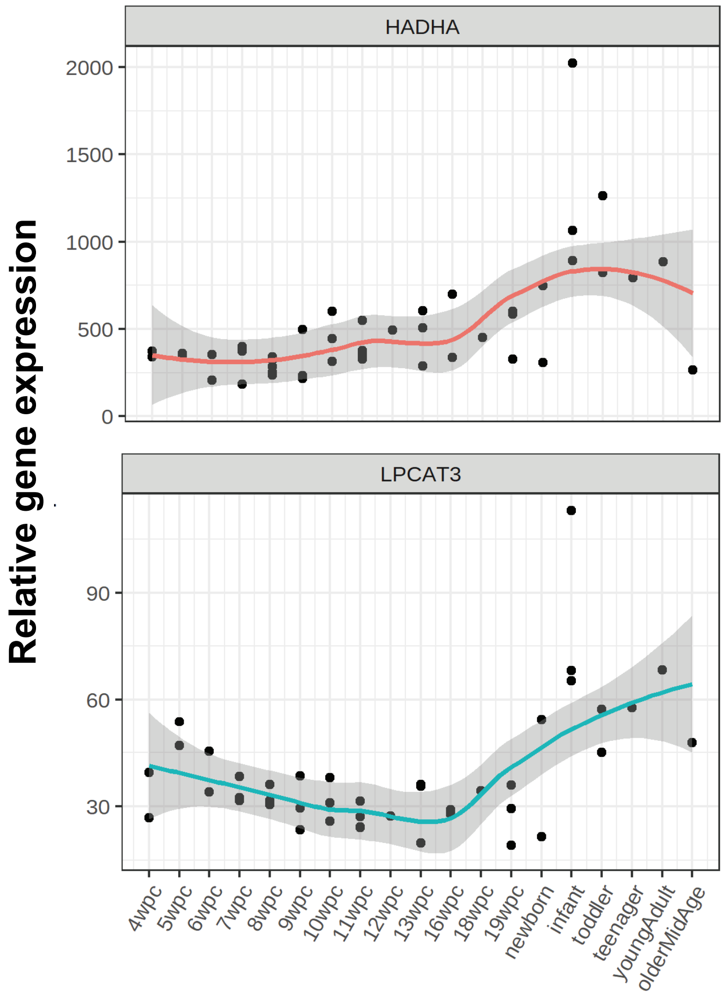

SUPPLEMENTAL FIGURE 9

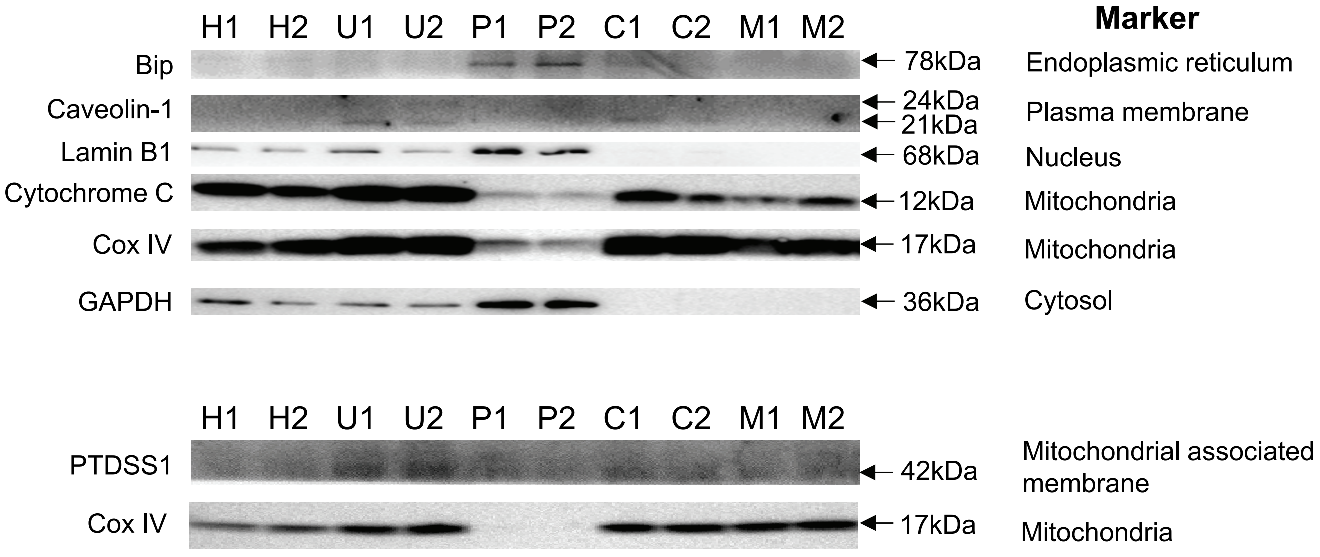

H: Homogenate
U: Unbroken cells and nuclei
P: Plasma membrane, lysosome, microsome and the cytosol
C: Crude mitochondria
M: Pure mitochondria

SUPPLEMENTAL FIGURE 10

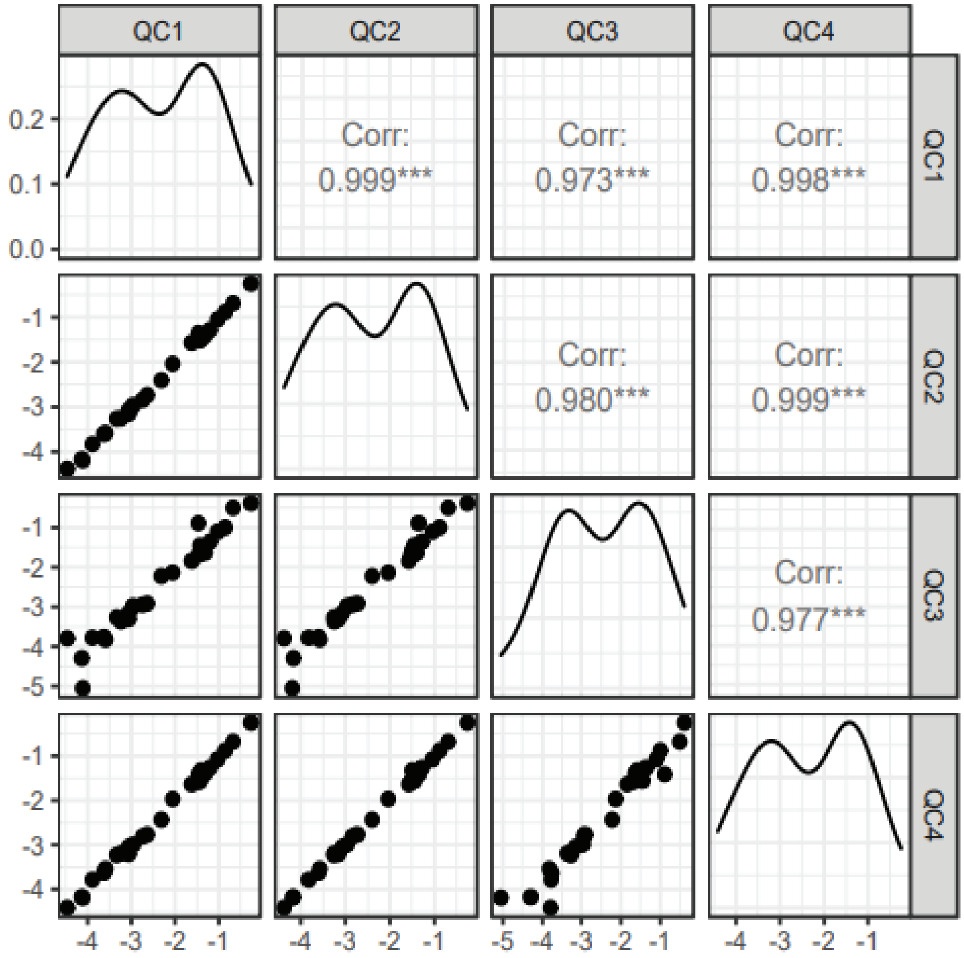

Supplement: Supplementary 1 — Fig. S1. Whole-heart lipidomes across cardiac development. Fig. S2. Distinct patterns of lipid changes across heart development. Fig. S3. Mitochondria lipidomes across cardiac development. Fig. S4. Enhanced mitochondria–lipid droplet contacts in postnatal cardiac development mediated by Plin-5. Fig. S5. Temporal changes of gene expression in the clusters determined by GPclust. Fig. S6. Temporal changes of lipid profiles in the clusters determined by GPclust. Fig. S7. Correlation of temporal changes between the clusters determined by GPclust of gene expression and lipids. Fig. S8. Relative gene expression levels of Hadha and Lpcat3 in human heart development. Fig. S9. Immunoblot assessment on the purity of crude mitochondria and pure mitochondria fractions isolated from P21 heart tissues. Fig. S10. Quality control across MS runs. [file research.0006.f1.pdf]
